# Autism phenotypes in ZnT3 null mice: Involvement of zinc dyshomeostasis, MMP-9 activation and BDNF upregulation

**DOI:** 10.1038/srep28548

**Published:** 2016-06-29

**Authors:** Min Heui Yoo, Tae-Youn Kim, Young Hee Yoon, Jae-Young Koh

**Affiliations:** 1 Neural Injury Research Lab, University of Ulsan College of Medicine, Seoul 138-736, Korea; 2 Department of Ophthalmology, University of Ulsan College of Medicine, Seoul 138-736, Korea; 3 Department of Neurology, University of Ulsan College of Medicine, Seoul 138-736, Korea

## Abstract

To investigate the role of synaptic zinc in the ASD pathogenesis, we examined zinc transporter 3 (ZnT3) null mice. At 4–5 weeks of age, male but not female ZnT3 null mice exhibited autistic-like behaviors. Cortical volume and neurite density were significantly greater in male ZnT3 null mice than in WT mice. In male ZnT3 null mice, consistent with enhanced neurotrophic stimuli, the level of BDNF as well as activity of MMP-9 was increased. Consistent with known roles for MMPs in BDNF upregulation, 2.5-week treatment with minocycline, an MMP inhibitor, significantly attenuated BDNF levels as well as megalencephaly and autistic-like behaviors. Although the ZnT3 null state removed synaptic zinc, it rather increased free zinc in the cytosol of brain cells, which appeared to increase MMP-9 activity and BDNF levels. The present results suggest that zinc dyshomeostasis during the critical period of brain development may be a possible contributing mechanism for ASD.

Autism spectrum disorder (ASD) is a pervasive neurodevelopmental disorder with characteristic symptoms such as deficits in social communication and interaction, difficulty in verbal and nonverbal communication^1^, and stereotyped behaviors such as body rocking and hand flapping^2^. In most ASD cases, autistic symptoms manifest at approximately 1–3 years of age^3^, indicating that the disease process starts during early childhood. One baffling fact is that the prevalence of ASD seems to be rapidly increasing; the most recent data from the Centers for Disease Control and Prevention report that the prevalence of ASD may be as high as 1 in 68 in the USA (CDC 2014). In addition, the prevalence of ASD in certain areas of South Korea may be as high as 2.64% and is therefore more than two times as prevalent as in the United States of America (USA)^4^. Although heightened awareness of ASD by clinicians and parents may be a contributing factor for this rise, it is likely that certain unidentified factors are also causing a real increase in ASD incidence worldwide. Whichever is the case, the current high prevalence is a considerable clinical and socioeconomic challenge, especially because there are no effective preventive or therapeutic measures that can fundamentally change the course of the disease.

Diverse causes of ASD - genetic, epigenetic, and environmental - have been identified, but common pathological mechanisms as a whole are not yet completely understood. Because more than 200 genes have been identified that are potentially linked to ASD^5^,^6^, it is likely that a second hit during the critical period of brain development may play an important role in causing ASD^7^. Some environmental factors, including infections, and prenatal or early postnatal nutritional deficiency, have been proposed as contributing conditions^8^,^9^. It is therefore of interest that dietary zinc deficiency may induce autistic-like behaviors in mouse models^10^. Because the stabilization of the postsynaptic density protein Shank3, a gene that has been linked to ASD^11^,^12^, is induced by zinc^13^,^14^, zinc deficiency around synapses may contribute to abnormal synaptic physiology in these mice. Alternatively, because zinc is involved in a number of critical changes during brain development^15^, zinc deficiency in the brain might cause structural changes in the brain that in some cases might lead to autism phenotypes^10^,^16^. In fact, the plasma zinc/serum copper ratio and hair zinc levels were reported to be decreased in ASD children^17^,^18^, which suggest that infantile zinc deficiency may be a risk factor for ASD.

One intriguing and unanswered feature of ASD is that brain growth during early childhood may be accelerated in certain cases of ASD. Kanner first noted macrocephaly in some ASD patients^19^. Since then, other investigators have reported similar findings^20^. One interesting point is that the head circumferences of ASD patients are normal at birth, but they became larger between 6 and 14 months of age^21^. MRI studies have shown that large head size is correlated with large brain size^22^. Consistent with these findings, the brains of ASD patients have been found to contain a larger number of neurons^6^. This increase in brain size appears to be age-dependent. Courchesne *et al*., for example, reported that increased brain volume may occur largely during early childhood, followed by normalization to control values at older ages^23^. The megalencephaly in ASD suggests that ASD brains may be developing under an altered trophic environment or altered developmental control. Levels of the neurotrophic factor BDNF are often increased in various models of ASD^24^ and may underlie brain overgrowth.

Considering these findings, we proposed the hypothesis that BDNF upregulation may be a core pathogenic mechanism for megalencephaly and autism phenotypes^24^. Previously, we demonstrated that MMP activation can lead to increased BDNF release and TrkB activation in cortical neuronal cultures^25^. Conditions that raise MMP activity in the brain may, in certain cases, lead to a phenotype that includes a larger brain and autistic-like behavior. Because mammalian brains contain a large and dynamic pool of zinc in glutamatergic synaptic vesicles (synaptic zinc), it is plausible that synaptic zinc plays a role in MMP activation and BDNF upregulation around synapses. However, contrary to our expectations, a recent study by Helgager demonstrated that ZnT3 null mice, in which synaptic zinc is absent, show increased levels of BDNF^26^. Hence, the absence of synaptic zinc, especially during brain development, may cause the upregulation of BDNF by an unidentified mechanism and may possibly induce large brain size and autistic phenotypes. In the present study, we used ZnT3 null mice to examine these possibilities.

## Results

### Male ZnT3 null mice exhibit autistic behavior

**Figure 1:**
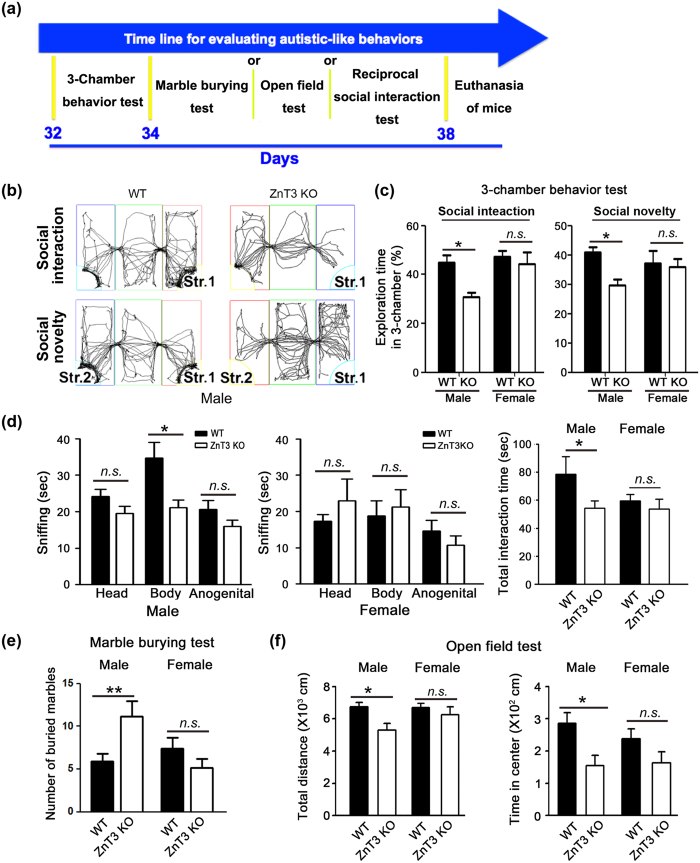
Autistic-like behaviors of male ZnT3 null mice. (**a**) A time-table to evaluate the autistic behavior in ZnT3 null or WT mice. The order for the behavior tests is 3-chamber social behavior test at PND32, marble burying test at PND34, and reciprocal social interaction test or open field test at PND36. (**b**) Tracking of the movements of mice in 3-chambered boxes. Male ZnT3 null mice moved less and interacted less with stranger 1 (social interaction test) and stranger 2 (social novelty test) than the male WT mice. (**c**) Bars denote the percent of exploration time in the chamber for stranger 1 (social interaction) or stranger 2 (social novelty) by male (right group) and female (left) WT and ZnT3 null mice (mean ± SEM, WT male, n = 24; KO male, n = 37; WT female, n = 11; KO female, n = 12, ***p < 0.001, *n.s*.: *p* < 0.05; two-way ANOVA with Bonferroni’s *post hoc* test). Female WT and ZnT3 KO mice showed little difference in either test, whereas male ZnT3 KO mice spent significantly less time in the stranger chambers in both tests. (**d**) Reciprocal social interaction test. Compared with male WT mice, the ZnT3 null mice exhibited diminished total sniffing time. By contrast, female mice showed no difference compared with WT in total interaction time (mean ± SEM, WT male, n = 18; KO male, n = 28; WT female, n = 11; KO female, n = 14, **p < 0.01, *n.s*.: *p* > 0.05; one-way ANOVA with Tukey’s *post hoc* test, three-way repeated measures ANOVA). (**e**) Repetitive behavior: Marble-burying test. Bars indicate that the male ZnT3 null mice buried more marbles than male WT mice (mean ± SEM, WT male, n = 32; KO male, n = 18; WT female, n = 14; KO female, n = 13, **p < 0.01, *n.s*.: *p* > 0.05; two-way ANOVA with Bonferroni’s *post hoc* test). (**f**) Open field test. Bars show the total distance moved and the time spent in the center. Male ZnT3 null mice moved less and spent less time in the center than male WT mice (mean ± SEM, WT male, n = 14; KO male, n = 15; WT female, n = 15; KO female, n = 10, *p < 0.05, *n.s*.: *p* > 0.05; one-way ANOVA with Tukey’s *post hoc* test, two-way ANOVA with Bonferroni’s *post hoc* test).

To evaluate the autistic behavior in ZnT3 null or WT mice, behavior tests were carried out in a genotype-blinded manner with the following order, 3-chamber social behavior test (postnatal day or PND32), marble burying test (PND34), open field and reciprocal social interaction test (PND36) (Fig. 1a). Social behaviors of zinc transporter 3 (ZnT3) null and wild-type (WT) mice were quantitatively investigated with 3-chamber tests. At 4 weeks of age, with experimenters blinded to genotypes, mice were placed in a 3-chambered box, and their behaviors were recorded with a computer-based motion analysis system. In the social interaction test, time spent in the empty chamber vs time spent in the chamber containing a stranger mouse (stranger 1) was measured. Compared with WT mice, male ZnT3 null mice spent more time in the empty chamber and less time in the chamber containing stranger 1 (Fig. 1b,c). The pattern observed in male ZnT3 null mice was consistent with reduced social interaction. Next, when a new stranger mouse (stranger 2) was placed in another chamber, male ZnT3 null mice tended to interact more with the now familiar stranger 1 than with stranger 2 (Fig. 1a,b), which indicated that novelty-seeking behavior was reduced in male ZnT3 null mice. By contrast, female ZnT3 null mice were not different in social interaction and social novelty behaviors from WT controls of the same sex (Fig. 1c). Next, a test for reciprocal social interactions was conducted. Overall interacting time and total interaction time were significantly shorter in male ZnT3 null mice compared with WT mice (Fig. 1d). By contrast, regional and total interaction times in female ZnT3 null mice were not different from WT female mice.

Next, we conducted marble burying tests to examine repetitive behavior. Compared with male WT mice, male ZnT3 null mice exhibited increased marble burying activity (Fig. 1e). Female mice showed little difference between the two groups (Fig. 1e). These data indicate that ZnT3 null mice tend to show more repetitive behavior in a male gender-specific fashion. In the open field test, male ZnT3 null mice spent significantly less time in the center of the cage than WT mice, another feature that is consistent with autistic-like behavior^27^. In addition, the total moving distance of male ZnT3 null mice was less than that of male WT mice (Fig. 1f). Although these mice appear to have normal motor strength, a more formal test may be needed to rule out the possible contribution by subtle motor weakness herein.

Neither total moving distance nor time spent in the center area was different in the female groups (Fig. 1f). However, no significant differences were noted between female WT and female ZnT3 null mice in all behavior tests. Thus, having determined that autistic phenol types occurred in only the male ZnT3 null mice, subsequent experiments were conducted in male mice.

### Brain size, neurite density, and neuronal numbers are increased in male ZnT3 null mice

**Figure 2:**
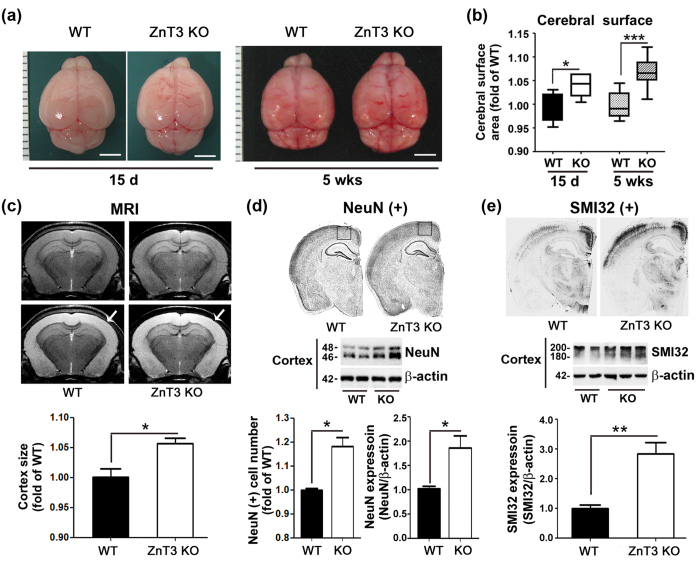
Increased brain size, neurite density and neuronal number in male ZnT3 null mice. (**a**) Photographs of representative brains from male WT and ZnT3 KO mice at 15 days and 5 weeks of age. Scale bars represent 3 mm. (**b**) Bars indicate the fold increase in cerebral surface as the area measured on an image analyzer (mean ± SEM, WT-15 d, n = 6; KO-15 d, n = 6; WT-5 wks, n = 21; KO-5 wks, n = 16, ***p < 0.001; two-way ANOVA with Bonferroni’s *post hoc* test with female cerebral surface). Although no difference was noted at 8 days of age, at 15 days of age and 5 weeks of age, male ZnT3 mice had larger brains than WT mice. (**c**) MRI for the measurement of cortex size. For the analysis, eight coronal slices from bregma +0.42 mm to −3.58 mm with a 0.5 mm interval were measured and analyzed using Image J software. Cortex size (whitened areas, arrows) of ZnT3 null brains was significantly larger than those of WT brains (mean ± SEM, WT, n = 8; KO, n = 10, *p < 0.05; Unpaired t-test with Welch’s correction). (**d**) Photographs of NeuN-stained coronal sections of brains of male WT and ZnT3 null mice. The cortical thickness of ZnT3 null brains was greater than in WT brains. And NeuN expression in the cortex of ZnT3 null brain was greater than that in WT brains. Bars denote the number of NeuN (+) neurons in the corresponding cortices (squares in D) and densitometry analysis of NeuN expression in the cortex of male WT and ZnT3 null brains (mean ± SEM, WT, n = 5; KO, n = 7, *p < 0.05; Unpaired t-test with Welch’s correction). (**e**) Photographs of male WT and ZnT3 null brains immunohistochemically stained for the neurofilament protein SMI-32. Western blot analysis showed the level of SMI-32 expression was substantially higher in the ZnT3 null cortex (mean ± SEM, WT, n = 8; KO, n = 10, ***p < 0.001; Unpaired t-test with Welch’s correction).

Postmortem examinations of brains revealed that at 5 weeks of age, the brains of male ZnT3 null mice were clearly larger in size than those of male WT mice (Fig. 2a,b). Although no difference was noted at 8 days of age (Fig. S1a,b), the size difference was evident and statistically significant even at 15 days of age, and it became greater at 5 weeks of age. At 5 weeks of age, the frontal region in particular appeared substantially larger in male ZnT3 null mice than in WT mice; as a result, their brains had a more roundish appearance (Fig. 2a). The cerebral surface areas of female ZnT3 null mice were not larger than those of female WT mice (Fig. S1c,d). To estimate cortical sizes in living mice, T2-weighted MRIs of brains were obtained. Cortical sizes were found to be greater in male ZnT3 null mice than in WT mice (Fig. 2c). NeuN immunostaining revealed that the number of neurons in the frontal area was larger and western blotting determined expression of NeuN was significantly increased in cortex of ZnT3 null brains than in WT brains (Fig. 2d). Finally, SMI32 (Neurofilament H Non-Phosphorylated) immunostaining revealed a marked increase in the density of SMI32 (+) neural processes in the cortical layers of male ZnT3 null mice. Consistently, SMI32 expressions in cortices of male ZnT3 null mice were significantly increased in Western blot analysis (Fig. 2e). Hence, male ZnT3 null mouse brains appeared to have undergone increased neurogenesis and neurite outgrowth during the early developmental stage, which raises the possibility that male ZnT3 null brains were developing in an environment with increased neurotrophic influence.

### Levels of BDNF and TrkB are increased in male ZnT3 null mice

**Figure 3:**
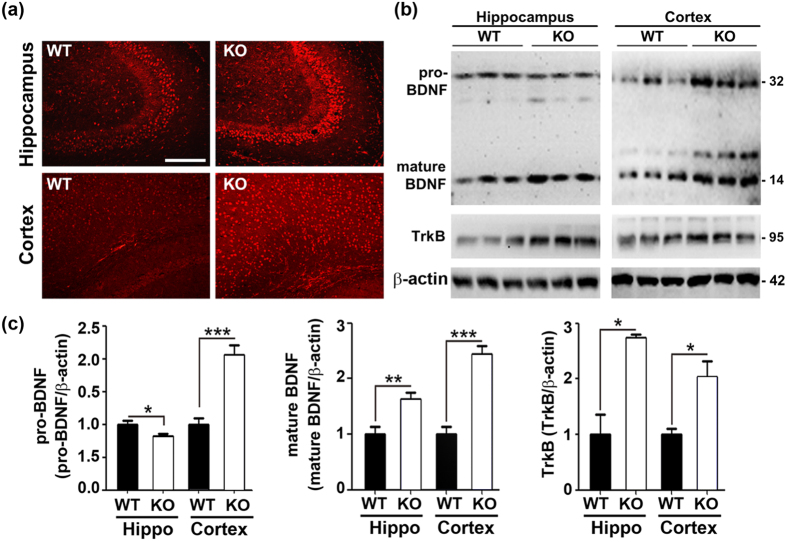
Increases in BDNF and TrkB levels in ZnT3 null mouse brains. (**a**) Fluorescence confocal photomicrographs of brains from male WT and ZnT3 null mice that were immune-histochemically stained for BDNF. BDNF immune-reactivity was substantially increased in ZnT3 null brains. Scale bar represents 200 μm. (**b**) Western blots of samples prepared from the hippocampus and neocortex of male WT and ZnT3 null mouse brains analyzed with antibodies to BDNF, TrkB, and β-actin. (**c**) Bars denote fold increases in the density of bands for pro-BDNF, mature BDNF, and TrkB in the hippocampus and cortex. Whereas mature BDNF and TrkB levels were increased in both regions in ZnT3 null brains compared with WT brains, pro-BDNF levels were increased in cortex, but not in the hippocampus (mean ± SEM, pro-BDNF: WT hippocampus (WT-H), n = 8; KO-H, n = 10; WT cortex (WT-CTX), n = 8; KO-CTX, n = 9, mature BDNF: WT-H, n = 8; KO-H, n = 10; WT-CTX, n = 12; KO-CTX, n = 16, TrkB: WT-H, n = 3; KO-H, n = 3; WT-CTX, n = 6, KO-CTX, n = 4, *p < 0.05, **p < 0.01, ***p < 0.001; two-way ANOVA with Bonferroni’s *post hoc* test with female results).

Consistent with this possibility, in several models of ASD such as a fragile X syndrome model (Fmr1−/−) of mice and valproate-treated mice, levels of the neurotrophic factor BDNF are increased^28^,^29^. Hence, we examined BDNF levels in maleZnT3 null mouse brains. Fluorescence immunohistochemical staining with an anti-BDNF antibody revealed that overall BDNF levels were significantly increased in male ZnT3 null brains compared with WT brains (Fig. 3a). Western blot analysis of cortical tissues also showed that ZnT3 null brains contained higher levels of both the pro-form and the mature form of BDNF in cortex. In hippocampus, only the mature form was increased. Furthermore, the level of TrkB, the main BDNF receptor, also appeared to be increased in ZnT3 null brains (Fig. 3b,c). Levels of NT4/5, NGF, and p75NTR were not altered (Fig. S2).

### Intracellular free zinc levels and MMP activity are paradoxically increased in ZnT3 null mouse brains

**Figure 4:**
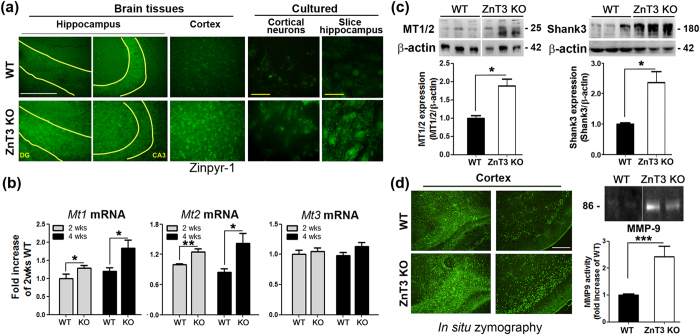
Paradoxical increases in intracellular free zinc levels and MMP activity in ZnT3 null mouse brains. (**a**) Fluorescence micrographs of neocortex in male WT and ZnT3 null brains, primary cortical neuronal cultures and hippocampal slice cultures stained for Zinpyr-1. Scattered faint zinc fluorescence was observed in WT cortices, whereas more diffuse and intense fluorescence was observed in ZnT3 null cortices and hippocampi. Yellow curved lines delineate dentate gyrus (DG) and CA3 regions of hippocampus. White scale bar represents 200 μm. Yellow scale bars represent 20 μm. (**b**) Quantification by real-time PCR of metallothione in levels in brain cortices. Whereas the level of *Mt3* did not differ between WT and ZnT3 null brains, the level of *Mt1* was substantially higher in ZnT3 null brains than in WT brains, which is consistent with the increase in free zinc levels observed in the former. *Mt1* mRNA levels in 4-week-oldZnT3 null brains was significantly increased compared with 2-week-old ZnT3 null brains (mean ± SEM, WT, n = 6, KO, n = 6 *p < 0.05, **p < 0.01; one-way ANOVA with Tukey’s *post hoc* test). (**c**) MT1/2 protein and Shank3 expression levels were significantly increased in the cortex of ZnT3 null mice compared with WT brains (mean ± SEM, MT1/2: WT, n = 5; KO, n = 5, Shank3: WT, n = 7; KO, n = 7, **p < 0.01; Unpaired t-test with Welch’s correction). (**d**) Fluorescence photomicrographs and MMP zymograms of proteins from male WT and ZnT3 null brains. Note that MMP (+) cells are more numerous in the ZnT3 null brains. Gel zymograms for MMP-9. Samples from ZnT3 null brains showed markedly increased MMP activity. Bars denote fold increase in MMP-9 activity in ZnT3 null brains compared with WT brains (n = 1) (mean ± SEM, WT, n = 7, KO, n = 6, ***p < 0.001; Two-way ANOVA with Bonferroni’s *post hoc* test with female MMP-9). Scale bar represents 200 μm.

In our previous study, we showed that zinc can activate MMPs and lead to BDNF release and TrkB activation in cultured cortical neurons^25^. Hence, we explored whether the ZnT3 null state might, in addition to resulting in the disappearance of zinc in synaptic vesicles, somehow alter free zinc levels in the cytosol of developing neurons. To assess this trait, we performed Zinpyr-1 staining. The results were contrary to our expectation that the ZnT3 null state would lead to decreased free zinc levels: we observed only faint zinc fluorescence that was localized in certain cell bodies in WT brains, cultured cortical neurons and slice cultured hippocampus, but zinc fluorescence in ZnT3 null brains, cultured cortical neurons and slice hippocampus appeared to be more intense and more diffuse (Fig. 4a). Consistent with increased free zinc loads in male ZnT3 null brain cells, levels of metallothioneins 1 and 2 (*Mt1/2*), which are known to be induced by increases in cytosolic free zinc levels, were found to be substantially increased in ZnT3 null brains (Fig. 4b,c). In contrast, mRNA levels of *Mt3* which is constitutively expressed regardless of cytosolic free zinc levels, did not change (Fig. 4b). Shank3 expression, modulated by zinc, was also significantly increased in the cortices of ZnT3 null mice (Fig. 4c). Next, we examined whether MMP levels and activity were altered in male ZnT3 null brains. Compared with WT brains, male ZnT3 null brains exhibited more intense staining in MMP zymograms (Fig. 4d). For instance, the superficial layers of the cortex as well as the corpus callosum showed increased MMP activity. Zymogram on protein electrophoresis gels also showed significant increases in the activity of MMP-9 in the male ZnT3 null brains compared with the WT brains (Fig. 4d). However, MMP activity was not altered in female ZnT3 null mice (Fig. S4a,b).

### Increases in *BDNF*, *Mt1*/*2* mRNA, BDNF/TrkB, and neurite outgrowth in cultured ZnT3 null cortical neurons

**Figure 5:**
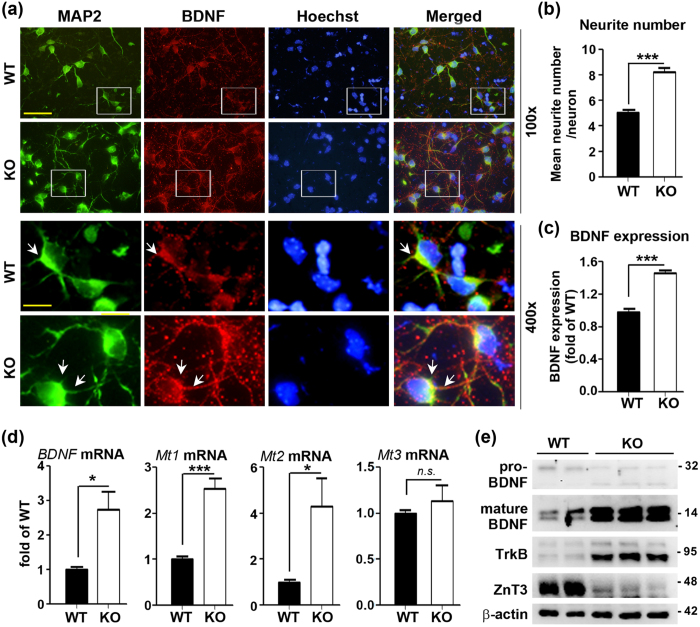
Increased neurite number and BDNF expression in cultured ZnT3 null neurons. **(a**) Fluorescence photomicrographs of WT and ZnT3 null neurons in culture, after immunocytochemical staining with anti-MAP2 and anti-BDNF antibodies. MAP2 and BDNF immunostaining intensities were increased in ZnT3 null neurons as compared with WT neurons. Arrows indicate 1^st^ branches of neurites. Scale bars represent 40 (upper) and 10 μm (lower). (**b**) The number of neurites was increased in cultured ZnT3 null neurons as compared with WT neurons (mean ± SEM, WT, n = 4; KO, n = 6, ***p < 0.001; Unpaired t-test with Welch’s correction). (**c**) Densitometric analysis of BDNF expression also showed that cultured ZnT3 null neurons contained higher levels of BDNF than WT neurons (mean ± SEM, WT, n = 4; KO, n = 6, ***p < 0.0001; Unpaired t-test with Welch’s correction). (**d**) Quantified real time PCR values for *BDNF*, *Mt1, 2* and *3* mRNA in samples of cultured WT and ZnT3 null mouse cortical neurons. *BDNF*, *Mt1*, and *2* mRNA levels were increased in cultured ZnT3 null cortical neurons (mean ± SEM, WT, n = 4; KO, n = 6, *p < 0.05, ***p < 0.001; Unpaired t-test with Welch’s correction). However, the level of *Mt3* mRNA showed no difference. (**e**) Western blot analysis of BDNF, TrkB, and ZnT3. The levels of mature BDNF and TrkB were increased in cultured ZnT3 null cortical neurons. All data were determined from three to four different sister culture for statistical comparisons.

Fluorescence immunocytochemical staining with an anti-MAP2 antibody revealed that the neurite number was significantly increased in cultured cortical neurons from ZnT3 null mice (Fig. 5a,b). Neurite outgrowth was observed in the cultured ZnT3 null cortical neurons (arrows). Moreover, fluorescence immunostaining with an anti-BDNF antibody appeared that BDNF expression was increased in neuronal bodies and dendrites of cultured ZnT3 null neurons (arrows), which consistent with BDNF expression in ZnT3 null brain tissue (Fig. 5a). Densitometric analysis of BDNF expression also showed that cultured ZnT3 null neurons contained higher levels of BDNF than WT neurons (Fig. 5c). Hence, cultured ZnT3 null neurons also appeared to have undergone increased neurite outgrowth with increased neurotrophic influences. Based on quantified real time PCR analysis, *Mt1/2* and *BDNF* mRNA levels were increased in the cultured ZnT3 null cortical neurons compared with WT neurons (Fig. 5d). The fact that *Mt1/2* mRNA levels were substantially increased, were consistent with possible increases in cytosolic free zinc levels in ZnT3 null cortical neurons. Again, the level of *Mt3* mRNA showed no difference between WT and KO cortical neurons in culture (Fig. 5d). These were consistent with the results obtained in WT and ZnT3 null mouse brains. Additionally, mature BDNF and TrkB levels were markedly increased in cultured ZnT3 null cortical neurons (Fig. 5e).

### Raising intracellular zinc levels increases MMP activity and BDNF expression

**Figure 6:**
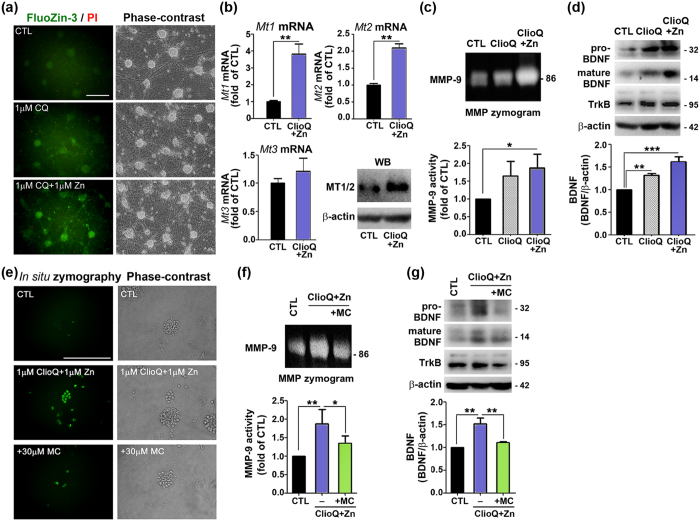
Raising intracellular zinc levels increases MMP-9 activity/BDNF expression, and minocycline blocks this effect in mixed cortical cultures. (**a**) Fluorescence photomicrographs of cortical cultures loaded with Fluozin-3-AM or PI with 1 μM ClioQ or ClioQ plus 1 μM zinc treatment for 1hour. Intracellular free zinc levels were substantially increased by treatment with ClioQ or ClioQ plus zinc without significant cell death (n = 3). Scale bar = 200 μm. (**b**) *Mt1/2* mRNA and protein levels were increased in the cultures with 1 μM ClioQ plus 1 μM zinc for 14 hours (mean ± SEM, n = 3, **p < 0.01; Unpaired t-test with Welch’s correction). However, *Mt3* was not increased. (**c**) The activity of MMP-9 was increased by ClioQ treatment. Bars denote densitometric values from MMP-9 zymogram bands (mean ± SEM, n = 5, *p < 0.05; One-way ANOVA with Tukey’s *post hoc* test). (**d**) The levels of pro-/mature BDNF and TrkB were increased in the cultures treated with same conditions. Bars denote fold increases in the density of mature BDNF bands (mean ± SEM, n = 3, **p < 0.01, ***p < 0.001; One-way ANOVA with Tukey’s *post hoc* test). (**e**) Fluorescence *in situ* MMP zymograms and matched phase-contrast photomicrographs of cortical cultures that were sham washed (CTL) compared with those treated for 1 hour with 1 μM ClioQ or 1 μM Zn alone or in the presence of 30 μM minocycline (MC). Whereas ClioQ plus zinc increased MMP activity in neurons, MC blocked this effect. Scale bar = 200 μm. (**f**) Gel MMP zymogram showed that the marked increases in MMP-9 activity observe following treatment with ClioQ plus Zn were substantially inhibited by the addition of minocycline. Bars denote fold increases in MMP-9 activity, as measured by densitometry on gel zymogram under these conditions (mean ± SEM, n = 5, **p < 0.01, *p < 0.05; one-way ANOVA with Tukey’s *post hoc* test). (**g**) Western blot analysis of BDNF levels. Bars denote fold increases in BDNF levels (as measured by densitometry) under the above conditions (mean ± SEM, n = 3, **p < 0.01; one-way ANOVA with Tukey’s *post hoc* test). All data were determined from three to four different sister culture for statistical comparisons.

Because free zinc levels seemed to be increased in the hippocampus and cortex of ZnT3 null brains, we examined whether increasing intracellular zinc could activate MMP-9in cortical cell cultures. To increase intracellular zinc levels, we exposed cultured cortical neurons to 1 μM clioquinol (ClioQ), a zinc ionophore, or ClioQ plus 1 μM zinc (Zn) for 1 hour. Staining with Fluozin-3 revealed that ClioQ and, to a greater extent, ClioQ plus Zn, increased intracellular free zinc levels without significant neuronal cell death (few PI stained cells) (Fig. 6a). As we observed in brains, ClioQ plus Zn markedly increased *Mt1/2* mRNA levels without significantly altering *Mt3* mRNA levels (Fig. 6b). MMP gel zymograms revealed that ClioQ plus Zn markedly increased MMP-9 activity (Fig. 6c). ClioQ and ClioQ plus Zn significantly increased the pro and mature forms of BDNF as well as TrkB, as assessed by Western blot analysis (Fig. 6d).

### Minocycline prevents changes in mixed cortical cultures and male ZnT3 null mice

Finally, we investigated a possible causal link between MMP-9 activation and BDNF upregulation, increased brain size, and autism phenotypes in ZnT3 null mice. We used minocycline to inhibit MMP-9 in cultures and in mice. In cultured neurons and astrocytes, in the ClioQ plus Zn experiments, minocycline markedly attenuated the MMP-9 activation that was induced by increased intracellular zinc (Fig. 6e,f). Furthermore, minocycline was effective at reducing BDNF and TrkB up-regulation in these cells (Fig. 6g).

**Figure 7:**
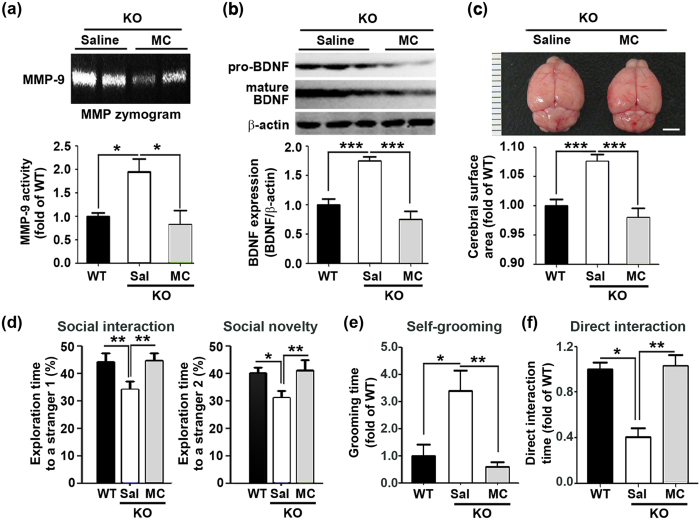
Minocycline reduces MMP activation, BDNF upregulation, and megalencephaly, and ameliorates autistic-like behaviors in male ZnT3 null mice. (**a**) MMP gel zymograms. Treatment with MC markedly reduced MMP-9 activity. Bars denote fold increases in MMP-9 activity, as measured by densitometry on these gels (mean ± SEM, WT, n = 6; KO-Saline, n = 7; KO-MC, n = 3, *p < 0.05; one-way ANOVA with Tukey’s *post hoc* test). (**b**) Western blot analysis of BDNF levels. Treatment with MC substantially reduced BDNF levels in male ZnT3 null brains. Bars denote fold increases in BDNF levels, as measured by densitometry on these gels (mean ± SEM, WT, n = 4; KO-Saline, n = 8; KO-MC, n = 6, *p < 0.05, **p < 0.01; one-way ANOVA with Tukey’s *post hoc* test). (**c**) Photographs show representative brains from male ZnT3 null mice that were saline-treated or MC-treated. Note that treatment with MC resulted in a reduction of brain size. Bars denote the size of the cerebrum (mean ± SEM, WT, n = 13; KO-Saline; n = 9, KO-MC; n = 8, **p < 0.01; one-way ANOVA with Tukey’s *post hoc* test). Scale bar = 3 mm. (**d**) Bars represent time spent with a stranger 1 or with a stranger 2 in the 3-chamber test. The interaction with stranger 1 was greater in 50 mg/kg MC-treated ZnT3 null mice than in saline-treated ZnT3 null mice. The interaction with stranger 2 was greater in the MC-treated group than in the saline treated ZnT3 null mice group (mean ± SEM, WT, n = 19; KO-Saline, n = 27; KO-MC, n = 14, *p < 0.05, **p < 0.01; one-way ANOVA with Tukey’s *post hoc* test). (**e**) Bars represent time spent on self-grooming in the 3-chamber test. Self-grooming of ZnT3 null mice was significantly increased compared with WT mice and it was reduced by MC administration (mean ± SEM, WT, n = 7; KO-Saline, n = 12; KO-MC, n = 8, *p < 0.05, **p < 0.01; one-way ANOVA with Tukey’s *post hoc* test). (**f**) Bars represent time spent in direct interaction with stranger 1 mouse in the 3-chamber test. The direct interaction time was greater in MC-treated ZnT3 null mice than in saline-treated ZnT3 null mice (mean ± SEM, WT, n = 6; KO-Saline, n = 10; KO-MC, n = 8, **p < 0.01; one-way ANOVA with Tukey’s *post hoc* test).

After confirming that minocycline acts *in vitro* as an effective MMP inhibitor, we tested it in ZnT3 null mice. Administration of 50 mg/kg minocycline three times per week for 2.5 weeks substantially reduced MMP-9 activity in male ZnT3 null brains (Fig. 7a). The rationale of choosing 7–27 days for the MC treatment was mainly because the growth spurt in brain size occurred during this early stage of development (5 weeks may be too late). Minocycline treatment also decreased BDNF levels in these mice (Fig. 7b). Furthermore, the brain size of male ZnT3 null mice was reduced by minocycline treatment to a size similar to normal control mice (Fig. 7c). Finally, the 3-chamber tests showed that minocycline treatment resulted in a reduction in autistic-like behaviors in male ZnT3 null mice (Fig. 7d–f). These results indicate that MMP activation contributed to the autism-related changes observed in ZnT3 null mice.

**Figure 8:**
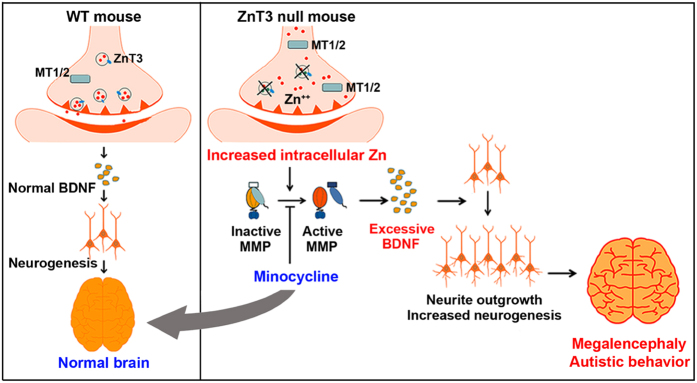
Abnormalities in Zinc-MMP-BDNF (ZMB) axis may contribute to the emergence of megalencephaly and autistic-like behaviors in ZnT3 null mouse. A diagram describing how the deletion of ZnT3 induces autistic-like behaviors with increased intracellular zinc and activated MMP-9 in ZnT3 null brains. Activated MMP-9 induces excessive BDNF expression and neurite outgrowth/neurogenesis. Autistic-like behaviors appeared in the male ZnT3 null mouse and were accompanied by megalencephaly. Minocycline administration ameliorated autistic-like behaviors in the male ZnT3 null mice by reducing MMP-9 activation and BDNF expression.

A diagram describes the mechanism by which minocycline reverses autistic behavior in the ZnT3 null mouse by decreasing MMP-9 activation and BDNF expression. Deletion of ZnT3 increases intracellular zinc and activates MMP-9 in the ZnT3 null brain via increased metallothionein-1/2. Activated MMP-9 induces excessive BDNF expression and neurite outgrowth/neurogenesis. Autistic-like behaviors appeared in ZnT3 null mice with megalocephaly. Minocycline administration ameliorated autistic-like behaviors in ZnT3 null mice by reducing the activation of MMP-9 and BDNF expression (Fig. 8).

## Discussion

The central finding of the present study is that male ZnT3 null mice exhibit features characteristic of ASD such as reduced social interactions and increased repetitive behavior relative to age-matched male WT mice. By contrast, ZnT3 null female mice exhibit no behavioral differences compared with female WT mice. This male gender specificity is consistent with the male preponderance in autism prevalence in humans^30^ and suggests an important role for the early influence of male sex hormones on aberrant brain development in this model.

Earlier studies of the role of zinc in brain development indicated that severe zinc deficiency during the prenatal period is associated with retarded brain growth and functional abnormalities^31^,^32^; in addition, overall neurogenesis is reduced, resulting in microcephaly. More recently, dietary zinc deficiency has been reported to cause autism phenotypes in mice, likely by reducing synaptic transmission^10^. Because the brain contains a dynamic pool of labile zinc, which is stored in synaptic vesicles through the action of ZnT3 (SLC30A3) and released upon depolarization to modulate synaptic physiology, it has been proposed that abnormalities in synaptic zinc levels may contribute to the above-mentioned brain dysfunctions such as microcephaly that are observed in nutritionally zinc-deficient animals^33^. Consistent with these ideas, dietary supplementation or injections of zinc have been shown to have beneficial effects in ASD patients^34^,^35^,^36^,^37^.

However, contrary to our original expectation, in the present study, the specific genetic deletion of ZnT3 in male mice resulted in the enlargement of the brain, and especially of the frontal region. Enlarged brains, or megalencephaly, may commonly accompany ASD in humans^19^,^38^. Although it is unclear whether neuronal and/or neurite overgrowth occurs in idiopathic ASD, functional MRI studies have suggested that most ASD brains may be hyperconnected^39^, a finding that is consistent with the increased neurite arborization noted in the present study. In fact, in a few animal ASD models that exhibit increased brain sizes, for instance, the *Pten* KO mouse model and valproate model, neuronal connectivity is also increased^5^,^40^. Consistent with these findings, the present results demonstrated that in male ZnT3 null mice, neuronal number, cortical thickness, and possibly neuronal size were all increased compared with age- and sex-matched WT mice. Although we did not examine the mice for functional hyperconnectivity in this study, the overall increase in SMI-32 (+) and MAP2 (+) neurite density seems to be consistent with the hyperconnectivity hypothesis. Thus far, there is no evidence that mutations or SNPs of the ZnT3 gene are linked to ASD. However, a recent study suggested that allelic variants of the ZnT3 gene are gender-specific, in this case female-specific, and are associated with schizophrenia, another neurodevelopmental disorder^41^. An interesting possibility is that certain SNP’s in the ZnT3 gene or other genes involved in zinc homeostasis might be found to be associated with ASD.

To search for possible biochemical causes of the observed change in brain size, we focused on BDNF, a potent neurotrophic factor, the upregulation of which has been implicated in certain cases of ASD^42^. In male ZnT3 null mice, the level of mature BDNF was increased in both the hippocampus and neocortex over levels observed in WT mice. Moreover, the level of pro-BDNF was increased in cortex but not in the hippocampus. Hence, although BDNF may be upregulated throughout the forebrain, its specific pattern of expression may differ between brain regions. Although the precise mechanism for BDNF upregulation in ZnT3 null mice is unknown, this finding is consistent with the report by Helgager that showed increased BDNF levels in relatively young ZnT3 null brains^26^. The combination of upregulated BDNF and increased brain size in male ZnT3 null mice suggests the interesting possibility that BDNF upregulation is one of the factors that causes large brains as well as autism phenotypes in these mice. Levels of TrkB, the tyrosine kinase receptor for BDNF that mediates most of its neurotrophic effects, were also increased in male ZnT3 null brains, whereas neither the levels of p75NTR, another neurotrophin receptor that favors the pro-from of BDNF, nor the levels of NGF and neurotrophin 4/5 were changed. BDNF and TrkB upregulation may be caused by zinc dyshomeostasis as seen in cultured cortical neurons exposed to clioquinol plus zinc. In addition to causing long-term increases in neurotropism, BDNF and TrkB may also alter short-term synaptic physiology as it modulates ion channels such as NMDA receptors^43^. BDNF phosphorylates NMDA receptors by activating Src and potentiating its function^44^. In fact, NMDA receptor hyperfunction has been implicated in the IRSp53 null mouse model of ASD, and an NMDA antagonist memantine reversed the autism phenotype in this model^45^.

How the BDNF upregulation is brought about in ZnT3 null mouse brains is not known. Because zinc itself can increase BDNF expression^46^, this finding in brains devoid of synaptic zinc was the opposite of our initial expectation. Another notable finding is that an enlarged brain and BDNF upregulation were observed by the time the mice were 15 days old, when the level of synaptic zinc is minimal in WT mice^47^. Hence, effect of the ZnT3 null state may not be due to the absence of synaptic zinc but to other unknown developmental changes caused by the absence of ZnT3. In fact, we found that zinc fluorescence, an indicator of free zinc levels, was substantially increased in cells in male ZnT3 null brains. Hence, it is possible that during the early developmental stage, the absence of ZnT3 may alter zinc homeostasis in non-vesicular compartments, including the cytosol. Such increases in free zinc levels in the cytosol may induce the upregulation of BDNF, as was shown in the results from cortical cell culture experiments. It is interesting that BDNF upregulation by zinc seems to be causally linked to MMP activation. In cultured neurons and astrocytes, the activation of MMPs increases BDNF levels, partly through induction and partly through proteolytic conversion of pro-BDNF to mature BDNF^25^,^48^. Our results showed that the rise in free zinc levels, as shown in male ZnT3 null brain cells and as induced in cultured cortical cells by raising cytosolic free zinc levels with clioquinol plus zinc, was associated with increased MMP levels and activity. The mechanism of MMP induction/activation by zinc warrants further investigation.

Although the precise mechanism that links ZnT3 deletion to autism remains to be determined, our results indicate that the upregulation of MMPs is critically involved. The inhibition of MMP activity with minocycline not only normalized the BDNF upregulation and increased brain size but also ameliorated autistic social behaviors in male ZnT3 null mice. The role of MMPs in ASD in general has not been extensively investigated. However, in an animal model of fragile X syndrome that is often associated with autism and macrocephaly^49^,^50^, MMP-9 activity in the brain was increased^51^ and MMP inhibition by minocycline reduces abnormal social behaviors in these mice^52^. Although clinical improvement was not obtained, minocycline treatment for 6 months did reduce cerebrospinal fluid (CSF) BDNF levels in 3–12 year-old patients^53^. The lack of effect on autism symptoms in this trial might have been due to the possibility that MMP activation and BDNF upregulation during the early stage may induce autism phenotypes, but BDNF upregulation at later stages may not play a large role in the maintenance of autism phenotypes. Despite the negative result on autism symptoms in this pilot trial, it may be useful to try minocycline at earlier time points of the disease, for instance, shortly after symptom onset. Since the MMP inhibitory effect of minocycline may also inhibit microglial activation and inflammation^54^, these effects may also mediate the beneficial effect of minocycline in male ZnT3 null mice.

It is intriguing that both zinc deficiency and zinc dyshomeostasis may be linked to ASD. While zinc deficiency may contribute to ASD through synaptic dysfunction, zinc dyshomeostasis during the critical developmental period may cause abnormal brain growth by the above mentioned pathway. Since megalencephaly may occur in a subset of ASD patients, zinc dyshomeostasis may be relevant in these cases.

Initially, we hypothesized that low levels of synaptic zinc would be the cause of ASD phenotype in ZnT3 null mice. However, what we found was, as described in the manuscript, rather substantial increases in free zinc levels in the cytosol of neurons in ZnT3 null mouse brains. That may explain why MMP activity and BDNF/TrkB levels were not decreased but increased. At this moment, it is unknown whether this mechanism – zinc dyshomeostasis, MMP activation, BDNF upregulation – operates in sporadic ASD cases, especially ones accompanied by megalencephaly. We hope that our study may provide a plausible and testable hypothesis in these human cases.

In conclusion, we propose the hypothesis that abnormalities in the zinc-metalloprotease-BDNF (ZMB) axis may be a common denominator mechanism that is involved in producing ASD associated with megalencephaly^24^. To further test this idea, we plan to utilize other genetic or toxin models of ASD and to examine the changes in synaptic zinc, MMP and BDNF in those models.

## Materials and Methods

### Experimental design

To evaluate the autistic behavior in ZnT3 null or WT mice, behavior tests were carried out with the following order, 3-chamber behavior, marble burying, open field and reciprocal social interaction test, in a blinded manner. After each trial, all chambers were cleaned with super hypochlorous water, an efficient odor removal agent with relatively weak odor, to prevent a bias based on olfactory cues.

### Animals

ZnT3 null mice and their wild-type (WT) littermates, hybrid of C57Bl/129Sv, were a generous gift from Professor Richard D. Palmiter at University of Washington. These mice were bred and maintained in the facility of the University of Ulsan, College of Medicine. Animals were allowed free access to food and water at 24 ± 0.5 °C and were exposed to a 12 hours light/dark cycle. All animal experiments were performed according to the Guidelines of the Institutional Animal Care and Use Committee (IACUC) at Asan Medical Center and Ulsan University College of Medicine.

### Materials

Anti-rabbit ZnT3 antibody was a kind gift from Professor Richard D. Palmiter (University of Washington, USA). Minocycline hydrochloride, Propidium Iodide (PI), and the anti-rabbit MAP2 were purchased from Sigma-Aldrich (USA). Anti-rabbit BDNF and anti-rabbit TrkB antibodies were obtained from Santa-Cruz Biotech. Inc. (USA). The anti-mouse NeuN antibody was obtained from Merck Millipore (USA). The anti-mouse SMI32 (Anti-Neurofilament H) antibody was obtained from Covance (Sternberger Monoclonal Inc., USA). All MMP zymogram materials were purchased from Thermo Fisher Scientific Inc. (USA).

### Minocycline-administration

Minocycline (50 mg/kg/10 ml)^55^ or the same volume of normal saline was given via the intraperitonial route to mice three times a week from postnatal day 7 to 27 in a blinded manner. The dosing schedule was adopted from the published paper^55^. The dosage of 50 mg/Kg was highly effective but with little toxic effect.

### 3-chamber social behavior test

Social interaction and social novelty tests were conducted as previously described^56^. Briefly, during the 10-minute habituation session, a subject was placed in the empty chamber 1 of the 3-chamber apparatus. During the second session (social interaction test, 10-minute), chamber 1 contained a stranger mouse (stranger 1) of the same sex and age that was enclosed in a wire jail, whereas chambers 2 and 3 were empty. The time the subject mouse spent in chamber 1, chamber 2 or 3 was measured using an automated tracking system with Smart 3.0 software (PanLab, Harvard Apparatus, Spain). During the third session (social novelty test, 10-minute), a second stranger mouse (stranger 2) was placed in chamber 3, enclosed in a wire jail, and the same procedure with the second section was followed. Self-grooming time and direct interaction with the stranger 1 during the second section were analyzed in a genotype and condition blinded manner.

### Reciprocal social interaction test

Reciprocal social interactions between two mice were measured as described by McFarlane^57^. A single mouse was allowed to freely explore a new environment for 10 minutes. An unfamiliar same strain/age male or female mouse was introduced to the cage and allowed to explore it freely for 10 minutes as a test session. All behaviors were video recorded and subsequently scored for behavioral events, including nose-to-head sniff, nose-to-body sniff and nose-to-anogenital sniff.

### Open field test

Total activity and center square exploration were assessed by placing the mouse in an empty open field box that was constructed of opaque plexi-glass (30 cm × 30 cm × 30 cm) for 30 minutes^27^. Time spent in the center square (25% of the total area) and total distance moved was measured using computer assisted tracking software (Smart 3.0 software, PanLab, Harvard Apparatus, Spain).

### Marble burying test

To assess the tendency towards repetitive behavior, a marble-burying test was conducted, as described by Thomas^58^. Before performing the experiments, all mice were habituated for 10 minutes to a cage (27.5 cm × 19.0 cm × 15.5 cm) that was filled with 4 cm of fresh corncob bedding. Then, 20 glass marbles (1.5 cm diameter, 4 × 5 arrays) were placed in the cage. The number of marbles buried (>50% marble volume covered by bedding material) in 10 minutes was recorded and counted for confirming repetitive behavior in mice^59^.

### Photographed brains and analyzed cerebral surface area

Immediately after sacrifice, whole brains of WT and ZnT3 null mice were photographed with a Canon Digital camera (Canon USA, Inc., USA) together with a scale bar. Areas of the photographed brains were measured with Image J software (NIH, USA) and each value was normalized to the mean value in same aged WT mice^60^.

### Magnetic resonance imaging (MRI) of mouse brains

All images were captured using a 9.4T/160 mm animal MRI system (Agilent Technologies, USA). Radio frequency excitation and all signal detections were performed with one 72 mm quadrature volume coil and two channel-phased array coils, respectively. The imaging protocol was for a T2-weighted image (T2WI) [(TR = 4000 ms; TE = 41.81 ms; slice number = 24; slice thickness = 0.5 mm; field of view = 16 × 16 mm^2^; and matrix = 256 × 256 (no gap)].

Volumetric analysis of MR images was performed using Image J software (NIH, USA). To analyze cortex volume, cortical area was measured with Image J software in eight coronal slices from bregma +0.42 mm to −3.58 mm with 0.5 mm intervals along the anteroposterior (AP) axis in T2WI. To measure mouse cortical volumes, all areas were calculated as area ×0.5 (as the height) and summed. Mouse cortical volumes were normalized to the mean of the WT mouse cortical volume.

### Zinc fluorescence

To detect free or labile zinc in brain tissues and cell cultures, Zinpyr-1 (Mellitech, ZP1, France) and FluoZin-3AM (Molecular Probes, Inc., USA) were used. Zinpyr-1 is a lipophilic fluorescent dye with a high specificity and affinity for Zn^2+^ (*K*_d_ = 0.7 ± 0.1 nM). Brain slides were incubated in 10 μM Zinpyr-1-containing PBS solution at 37 **°**C for 30 minutes. After thoroughly rinsing the slides with PBS, 10 μM Zinpyr-1 fluorescence was observed at a wavelength of 488 using a fluorescence microscope (BX60; Olympus, Japan) and a digital camera (DP70; Olympus, Japan). For detecting intracellular zinc in the cultures, each well was incubated in 5 μM FluoZin-3 AM containing minimum essential medium (MEM) at 37 **°**C for 30 minutes. And the following steps were same with Zinpyr-1 detection.

### Immunocyto- or histochemistry

For the immunocytochemistry and immunohistochemistry to detect BDNF, MAP2, NeuN and SMI32, brain tissues or primary cortical cell cultures on slides were fixed in 4% paraformaldehyde. Antibodies were diluted in PBS containing 2% bovine serum albumin (BSA) and then placed on the slides at 4 **°**C overnight. Alexa Fluor 488- or 555- donkey rabbit or mouse secondary antibodies (Invitrogen, Life Technologies, USA) were used for visualization. Finally, the obtained immunocytochemical fluorescence signals were visualized using a LSM710 confocal microscope (Zeiss, Germany) or a conventional fluorescence microscope (Olympus, Japan).

### Immunoblots

Equal amounts of protein were electrophoretically separated with 8–12% SDS-PAGE and transferred to PVDF membranes (Bio-Rad laboratories Inc., USA). Anti-rabbit-BDNF, rabbit-TrkB and anti-rabbit actin antibodies were diluted in 5% non-fat milk contained Tris-Buffered Saline and Tween 20 (TBST) and then placed on the membranes at 4 **°**C for overnight. Chemi-luminescent HRP substrate (Millipore, USA) was used to visualize the immune-reactive bands under the UVP Auto Chemi Darkroom Imaging System (Ultra-Violet Products Ltd, UK).

### Cortical cell cultures

Cortical astrocyte cultures were prepared from newborn mice as previously described^25^. After 2 weeks *in vitro* (DIV14), astrocyte cultures were used as feeder cultures for embryonic neuronal cells. Cortical neurons were obtained from embryonic mouse cortex of brains (E14) and were plated onto astrocyte cultures to generate mixed cortical cultures. For cortical pure neuronal culture of WT and ZnT3 null mouse, cortical neurons were collected from embryonic mouse (WT and ZnT3 null) brains (E14) and followed same processes as previously described^25^.

### MMP zymography

Lysates were quantified to attract equal amount of protein and then incubated with 50 μl Gelatin-Sepharose 4B (GE Healthcare, Sweden). Each pellet was washed with PMSF-containing PBS and then electrophoresed with 10% Zymogram gels (Invitrogen). The active form of MMP-9 (86 kD) was visualized on gels with Coomassie Blue staining. An Enz-Chek Gelatinase Assay Kit (Molecular Probes, USA) was used for *in situ* zymography by the manufacturer’s protocol.

### Real Time PCR

Total RNA was extracted from astrocytes using TRIzol reagent (Invitrogen, USA). A QuantiTect reverse transcription kit (Qiagen, Germany) was used to generate cDNA according to the manufacturer’s instructions. Samples were run in quadruplicate for 40–48 cycles using a 7300 Real Time PCR system (Applied Biosystems, USA). PCR was performed using SYBR Premix Ex Taq master mix (Takara Bio, Japan) and forward and reverse primers for *Mt1* (5′-CCTCTAAGCGTCACCACGACTTC-3′ and 5′-GGAGGTGCACTT GCAGTTCTTG-3′), *Mt2* (5′-CGCCTGCAAATGCAAACAAT-3′ and 5′-CTGCACTTG TCGGAAGCCTCT-3′), *Mt3* (5′-TGCAAATGCACGAACTGCAA-3′ and 5′-CCAGGG ACACCCAGCACTATTTAC-3′) and *BDNF* (5′-CCGGTATCCAAAGGCCAACTG-3′ and 5′-GGCATTGCGAGTTCCAGTGC-3′). Values were normalized to those of the housekeeping gene GAPDH (5′-GCCTCGTCCCGT AGACAAAAT-3′ and 5′-GTGACCAGGCGCCCAATA-3′).

### Statistics

Prism GraphPad (version 5) and SPSS were used to perform statistical analyses. In order to evaluate effects of multiple factors on various outcomes, we have applied two or three way analysis of variance (ANOVA) with two factor interactions. For comparing combination of multiple factors’ levels, multiple factors were re-parameterized to one factor of which level was combination of multiple factor levels, then the factor was evaluated one way ANOVA with multiple comparison. Significance level for multiple comparisons was adjusted using Tukey’s or Bonferroni’s method. Two-group comparisons were analyzed using an Unpaired t-test with Welch’s correction. A value of *p* < 0.05 was considered to represent a significant difference for all tests (Supplementary Table S1).

All experimental protocols were approved by Asan Institute for Life Sciences at Asan Medical Center and Ulsan University College of Medicine.

## Additional Information

**How to cite this article**: Yoo, M. H. *et al*. Autism phenotypes in ZnT3 null mice: Involvement of zinc dyshomeostasis, MMP-9 activation and BDNF upregulation. *Sci. Rep.* **6**, 28548; doi: 10.1038/srep28548 (2016).

## Supplementary Material

Supplementary Information
