# Supplementary material for: Autism phenotypes in ZnT3 null mice: Involvement of zinc dyshomeostasis, MMP-9 activation and BDNF upregulation
Source: Sci Rep. 2016 Jun 29;6:28548. doi: 10.1038/srep28548 (PMC4926223; doi:10.1038/srep28548)
Supplement: Supplementary Information [file srep28548-s1.doc]

**Autism phenotypes in ZnT3 null mice: Involvement of zinc dyshomeostasis, MMP-9**

**activation and BDNF upregulation**

Min Heui Yoo^a^, Tae-Youn Kim^a^, Young Hee Yoon^c^, Jae-Young Koh^a,b^*

^a^Neural Injury Research Lab, ^b^Department of Neurology and ^c^Department of Ophthalmology, University of Ulsan College of Medicine, Seoul 138-736, Korea

* To whom correspondence should be addressed:

Jae-Young Koh

Department of Neurology

University of Ulsan College of Medicine

388-1 Poongnap-Dong, Songpa-Gu

Seoul 138-736, Korea

Phone: 82-2-3010-4127

Fax: 82-2-483-5446

E-mail:[jkko@amc.seoul.kr](mailto:jkko@ amc.seoul.kr)

| **Supplementary Table S1. Statistical analysis** | | | | | |
| --- | --- | --- | --- | --- | --- |
| ***Znt3* mice BEHAVIOR** | | | | | |
| **Experiments** | **Parameter** | **Figure** | **Comparison** | **N** | **Statistical tests and significance** |
| 3-Chamber behavior test | Social interaction | Figure 1c | WT male (WT-M),  KO male (KO-M),  WT female (WT-F),  KO female (KO-F) | 24, 37, 11, 12 | Two-way ANOVA: Sex effect P= 0.0119, Gene effect P=0.0061 WT-M vs. KO-M, P < 0.001, WT-F vs. KO-F, n.s. (Bonferroni post test) |
|  |  |  |  |  | One-way ANOVA, WT-M vs KO-M, P<0.001, WT-M vs WT-F, n.s., WT-M vs KO-F, n.s., KO-M vs. WT-F, P<0.01, KO-M vs. KO-F, P<0.05, WT-F vs. KO-F, n.s. (Tukey's post test) |
|  | Social novelty | Figure 1c | WT male (WT-M),  KO male (KO-M), WT female (WT-F), KO female (KO-F) | 24, 37, 11, 12 | Two-way ANOVA: Sex effect P= 0.6957, Gene effect P=0.0269 WT-M vs. KO-M, P < 0.001, WT-F vs. KO-F, n.s. (Bonferroni post test) |
|  |  |  |  |  | One-way ANOVA, WT-M vs. KO-M, P<0.001, WT-M vs KO-M, n.s., WT-M vs WT-F, ns, WT-M vs KO-F, n.s., KO-M vs WT-F, n.s., KO-M vs KO-F n.s. (Tukey's post test) |
|  | Social interaction -minocycline (litters) | Figure 7d,  Suppl Figure S6a | WT litters-Saline (WT litters-S),  KO litters -Saline (KO litters-S),  KO litters -Minocycline (KO litters-M),  WT litters -Minocycline (WT-MC) | 19, 24, 14, 9 | Two-way ANOVA: Gene and Drug interaction effect P=0.0061, Gene effect P= 0.2047, Drug effect P=0.1552  WT-S vs. KO-S, P < 0.01, KO-S vs. KO-M, P<0.01, WT-S vs. WT-M, n.s. (Bonferroni post test) |
|  |  |  |  |  | One-way ANOVA, WT litters-S vs WT litters-M, n.s., WT litters -S vs KO litters -S, P<0.01, WT litters -S vs KO litters -M, n.s., WT litters -M vs KO litters-S, n.s., KO litters -S vs KO litters -M, P<0.01 (Tukey's post test) |
|  | Social interaction -minocycline  (dams) | Suppl Figure S6a | WT dams-Saline (WT dams-S),  KO dams-Saline (KO dams-S),  KO dams-Minocycline (KO dams-M), | 5, 6, 6 | One-way ANOVA, WT dams-S vs WT dams-M, n.s., WT dams -S vs KO dams-S, P<0.05, WT dams-S vs KO dams-M, n.s., KO dams-S vs KO dams -M, P<0.01 (Tukey's post test) |
|  | Social novelty -minocycline (litters) | Figure 7d, Suppl Figure S6b | WT litters-Saline (WT litters-S),  KO litters -Saline (KO litters-S),  KO litters -Minocycline (KO litters-M),  WT litters -Minocycline (WT-MC) | 19, 24, 14, 9 | Two-way ANOVA: Gene and Drug interaction effect P=0.0044, Gene effect P= 0.3797, Drug effect P=0.0588 WT litters-S vs. KO litters -S, P < 0.05, KO litters -S vs. KO litters-M, P<0.01, WT litters -S vs. WT litters-M, n.s. (Bonferroni post test) |
|  |  |  |  |  | One-way ANOVA, WT litters-S vs WT litters-M, n.s., WT litters -S vs KO litters -S, P<0.05, WT litters-S vs KO litters-M, n.s., WT litters-M vs KO litters-S, n.s., KO litters-S vs KO litters-M, P<0.01 (Tukey's post test) |
|  | Social novelty -minocycline  (dams) | Suppl Figure S6b | WT dams-Saline (WT dams-S),  KO dams-Saline (KO dams-S),  KO dams-Minocycline (KO dams-M), | 5, 6, 6 | One-way ANOVA, WT dams-S vs WT dams-M, n.s., WT dams -S vs KO dams-S, P<0.0001, WT dams-S vs KO dams-M, n.s., KO dams-S vs KO dams -M, P<0.0001 (Tukey's post test) |
|  | Self-grooming (litters) | Figure 7e,  Suppl Figure S6c | WT litters-Saline (WT litters-S),  KO litters -Saline (KO litters-S), KO litters -Minocycline (KO litters-M), | 7, 12, 8 | One-way ANOVA, WT litters-S vs. KO litters-S, P<0.05, WT litters -S vs. KO litters -M, n.s, KO litters -S vs. KO litters-M , P<0.01 (Tukey's post test) |
|  | Self-grooming (dams) | Suppl Figure S6c | WT dams-Saline (WT dams-S),  KO dams-Saline (KO dams-S),  KO dams-Minocycline (KO dams-M), | 3, 4, 4 | One-way ANOVA, WT dams-S vs. KO dams-S, P<0.01, WT dams -S vs. KO dams -M, n.s, KO dams -S vs. KO dams-M , P<0.01 (Tukey's post test) |
| 3-Chamber behavior test | Direct interaction  (litters) | Figure 7f, ,  Suppl Figure S6d | WT litters-Saline (WT litters-S),  KO litters -Saline (KO litters-S), KO litters -Minocycline (KO litters-M), | 6, 10, 8 | One-way ANOVA, WT litters-S vs. KO litters-S, P<0.05, WT litters -S vs. KO litters -M, n.s, KO litters -S vs. KO litters-M , P<0.01(Tukey's post test) |
|  | Direct interaction  (dams) | Suppl Figure S6d | WT dams-Saline (WT dams-S),  KO dams-Saline (KO dams-S),  KO dams-Minocycline (KO dams-M), | 3, 4, 4 | One-way ANOVA, WT dams-S vs. KO dams-S, P<0.05, WT dams -S vs. KO dams -M, n.s, KO dams -S vs. KO dams-M , P<0.01 (Tukey's post test) |
| Reciprocal social interaction test | Total interaction | Figure1d | WT male (WT-M) vs.  KO male (KO-M) WT female (WT-F) vs.  KO female (KO-F) | 18, 28, 11, 14 | Two-way ANOVA: Sex effect P= 0.1919, Gene effect P=0.0082 WT-M vs. KO-M, P < 0.05, WT-F vs. KO-F, n.s. (Bonferroni post test) |
|  |  |  |  |  | One-way ANOVA, WT-M vs. KO-M, p<0.05, WT-M vs. WT-F, p<0.01, WT-M vs. KO-F, p<0.05, KO-M vs. WT-F, n.s., KO-M vs. KO-F, n.s. (Tukey's post test) |
|  | Body region | Figure1d | Sex, Genotyping, and  Body reigon | 18, 28, 11, 14 | Three-way repeated measures ANOVA; main effect of sex:F=5.969, P=0.016; main effect of genotype:F=6.868, P=0.010; main effect of body region:F=5.319, P=0.006; main effect of genotypeXsex:F=10.647, P=0.001; main effect of body regionXsex:F=0.784, P=0.458; main effect of body regionXgenotype:F=1.614, P=0.202; main effect of body regionXgenotypeXsex:F=3.693, P=0.027 |
|  |  |  | WT-M vs. KO-M nose-nose (NN),  nose-body(NB),  nose-anogenital (NA) | 18, 28, 11, 14 | One-way ANOVA, WT-M-NN vs. KO-M-NN, n.s*.,*WT-M-NB vs. KO-M-NB, P < 0.01, WT-M-NA vs. KO-M-NA, n.s., WT-M-NN vs. WT-M-NB, n.s., WT-M-NN vs. WT-M-NA, n.s., WT-M-NN vs. KO-M-NB, n.s., WT-M-NN vs. KO-M-NA, n.s., WT-M-NB vs. WT-M-NA, P < 0.01, WT-M-NB vs. KO-M-NN, P < 0.001, WT-M-NB vs. KO-M-NA, P < 0.001, WT-M-NA vs. KO-M-NN n.s., WT-M-NA vs. KO-M-NB, n.s., KO-M-NN vs. KO-M-NB, n.s., KO-M-NN vs. KO-M-NA, n.s., KO-M-NB vs. KO-M-NA, n.s. (Tukey's post test) |
|  |  |  | WT-F vs. KO-F nose-nose (NN),  nose-body(NB),  nose-anogenital (NA) | 11, 14 | One-way ANOVA, WT-F-NN vs. KO-F-NN, n.s*.,*WT-F-NB vs. KO-F-NB, n.s, WT-F-NA vs. KO-F-NA, n.s., WT-F-NN vs. WT-F-NB, n.s., WT-F-NN vs. WT-F-NA, n.s., WT-F-NN vs KO-F-NB, n.s., WT-F-NN vs. KO-F-NA, n.s., WT-F-NB vs. WT-F-NA, n.s, WT-F-NB vs. KO-F-NN, n.s, WT-F-NB vs. KO-F-NA, n.s, WT-F-NA vs. KO-F-NN n.s., WT-F-NA vs. KO-F-NB, n.s., KO-F-NN vs. KO-F-NB n.s., KO-F-NN vs. KO-F-NA, n.s., KO-F-NB vs. KO-F-NA, n.s., n.s. (Tukey's post test) |
|  |  |  | WT-M-NN vs.  KO-M-NN WT-F-NN vs.  KO-F-NN | 18, 28, 11, 14 | Two-way ANOVA: Sex effect P= 0.7524, Gene effect P=0.3015 WT-M-NN vs. KO-M-NN, n.s., WT-F-NN vs. KO-F-NN, n.s. (Bonferroni post test) |
|  |  |  |  |  | One-way ANOVA, WT-M-NN vs. KO-M-NN, n.s, WT-M-NN vs. WT-F-NN, n.s., KO-M-NN vs. WT-F-NN, n.s., KO-M-NN vs. KO-F-NN, n.s. (Tukey's post test) |
|  |  |  | WT-M-NB vs.  KO-M-NB WT-F-NB vs.  KO-F-NB | 18, 28, 11, 14 | Two-way ANOVA: Sex effect P= 0.13628, Gene effect P=0.0094 WT-M-NB vs. KO-M-NB, P<0.01, WT-F-NB vs. KO-F-NB, n.s. (Bonferroni post test) |
|  |  |  |  |  | One-way ANOVA, WT-M-NB vs. KO-M-NB, P<0.05., WT-M-NB vs. WT-F-NB, P<0.01, WT-M vs KO-F, P<0.05, KO-M-NB vs. WT-F-NB, n.s., KO-M-NB vs. KO-F-NB, n.s. (Tukey's post test) |
|  |  |  | WT-M-NA vs.  KO-M-NA WT-F-NA vs. KO-F-NA | 18, 28, 11, 14 | Two-way ANOVA: Sex effect P=0.0632, Gene effect P=0.046 WT-M-NA vs. KO-M-NA, n.s, WT-F-NA vs. KO-F-NA, n.s. (Bonferroni post test) |
|  |  |  |  |  | One-way ANOVA, WT-M-NA vs. KO-M-NA, n.s., WT-M-NA vs. WT-F-NA, n.s., WT-M-NA vs. KO-F-NA, P<0.05, KO-M-NA vs. WT-F-NA, n.s., KO-M-NA vs. KO-F-NA, n.s., WT-F-NA vs. KO-F-NA, n.s. (Tukey's post test) |
| Marble burying test | Number of  buried marbles | Figure 1e | WT male (WT-M),  KO male (KO-M),  WT female (WT-F),  KO female (KO-F) | 32, 18, 14, 13 | Two-way ANOVA: Sex effect P= 0.1052, Gene effect P=0.2673 WT-M vs. KO-M, P < 0.01, WT-F vs. KO-F, n.s. (Bonferroni post test) |
|  |  |  |  |  | One-way ANOVA, WT-M vs. KO-M, P<0.05, WT-M vs. WT-F, n.s., WT-M vs. KO-F, n.s., KO-M vs. WT-F, n.s. KO-M vs. KO-F, P<0.05, WT-F vs. KO-F, n.s. (Tukey's post test) |
| Open field test | Total distance | Figure1f | WT male (WT-M) vs. KO male (KO-M) WT female (WT-F) vs. KO female (KO-F) | 14, 15, 15, 10 | Two-way ANOVA: Sex effect P= 0.2479, Gene effect P=0.0097 WT-M vs. KO-M, P < 0.01, WT-F vs. KO-F, n.s. (Bonferroni post test) |
|  |  |  |  |  | One-way ANOVA, WT-M vs. KO-M, P<0.05, WT-M vs. WT-F, n.s., KO-M vs. WT-F, P<0.05, KO-M vs. KO-F, n.s. (Tukey's post test) |
|  | Time in center | Figure1f | WT male (WT-M) vs. KO male (KO-M) WT female (WT-F) vs. KO female (KO-F) | 14, 15, 15, 10 | Two-way ANOVA: Sex effect P= 0.4739, Gene effect P=0.0034 WT-M vs. KO-M, P < 0.01, WT-F vs. KO-F, n.s. (Bonferroni post test) |
|  |  |  |  |  | One-way ANOVA, WT-M vs. KO-M, P<0.05, WT-M vs. WT-F, n.s., KO-M vs. WT-F, n.s., KO-M vs. KO-F, n.s. (Tukey's post test) |
| Cerebral surface area | Measuing cerebral  surface area | Figure 2b,  Suppl Figure S1 | WT male (WT-M),  KO male (KO-M),  WT female (WT-F),  KO female (KO-F) | 21, 16, 14, 7 | Two-way ANOVA: Sex effect P= 0.0078 Gene effect P=0.0355 WT-M vs. KO-M, P < 0.001, WT-F vs. KO-F, n.s. (Bonferroni post test) |
|  |  |  |  |  | One-way ANOVA, WT-M vs. KO-M, P<0.001, WT-M vs. WT-F, n.s., WT-M vs. KO-F, n.s., KO-M vs. WT-F, P<0.01. KO-M vs. KO-F, P<0.01, WT-F vs. KO-F, n.s. (Tukey's post test) |
|  |  |  | WT male-PND8, (WT-p8),  KO male-PND8 (KO-p8) | 5, 5 | Mann Whitney test, P = 0.2087, Unpaired t test with Welch's correction, P=0.1090 |
|  |  |  | WT male-PND15 (WT-p15),  KO male-PND15 (KO-p15) | 6, 6 | Mann Whitney test, P = 0.0411, Unpaired t test with Welch's correction, P=0.0346 |
|  |  |  | WT male-5wks (WT-5wks),  KO male-5wks (KO-5wks), | 21, 16 | Mann Whitney test, P <0.0001, Unpaired t test with Welch's correction, P<0.0001 |
|  |  | Figure 7c | WT -Saline (WT-S),  KO-Saline (KO-S),  KO-Minocycline (KO-M) | 13, 9. 8 | One-way ANOVA, WT-S vs KO-S, p<0.001, WT-S vs KO-M, n.s., KO-S vs KO-M, P<0.001 (Tukey's post test) |
| MRI | Measuing  cortex volume | Figure 2c | WT male (WT-M),  KO male (KO-M) | 8, 10 | Mann Whitney test, P = 0.0117, Unpaired t test with Welch's correction, P=0.0293 |
| Western blot | NeuN expression | Figure 2d | WT male (WT-M),  KO male (KO-M) | 5, 7 | Mann Whitney test, P = 0.0025, Unpaired t test with Welch's correction P=0.0155 |
|  | SMI32 expression | Figure 2d | WT male (WT-M), KO male (KO-M) | 8, 10 | Mann Whitney test, P< 0.0001, Unpaired t test with Welch's correction P=0.0008 |
|  | pro-BDNF expression -male only | Figure 3c | WT-male Hippocampus (WT-MH), KO-male Hippocampus (KO-MH) | 8, 10 | Mann Whitney test, P=0.0117, Unpaired t test with Welch's correction P=0.0168 |
|  |  |  | WT-male Cortex (WT-MC), KO-male Cortex (KO-MC) | 8, 9 | Mann Whitney test, P = 0.0003, Unpaired t test with Welch's correction P<0.0001 |
|  | mature BDNF expression- male only | Figure 3c | WT-male Hippocampus (WT-MH), KO-male Hippocampus  (KO-MH) | 8, 10 | Mann Whitney test, P=0.0044, Unpaired t test with Welch's correction, p=0.0024 |
|  |  |  | WT-male Cortex (WT-MC), KO-male Cortex (KO-MC) | 12, 16 | Mann Whitney test, P < 0.0001, Unpaired t test with Welch's correction P<0.0001 |
|  | TrkB expression -male only | Figure 3c | WT-male Hippocampus (WT-MH), KO-male Hippocampus (KO-MH) | 3, 3 | Mann Whitney test, P=0.10, Unpaired t test with Welch's correction, P=0.042 |
|  |  |  | WT-male Cortex (WT-MC), KO-male Cortex (KO-MC) | 6, 4 | Mann Whitney test, P =0.0095, Unpaired t test with Welch's correction, P=0.0447 |
|  | pro-DNF expression -male and female | Figure 3c | WT male cortex (WT-MC), KO male cortex (KO-MC), WT female cortex (WT-FC), KO female cortex (KO-FC) | 8, 9, 5, 7 | Two-way ANOVA: Sex effect p<0.0001, Gene effect P=0.0008 WT-MC vs. KO-MC, P <0.0001 , WT-FC vs. KO-FC, n.s. (Bonferroni post test) |
|  |  |  |  |  | One-way ANOVA, WT-MC vs KO-MC p<0.001, WT-MC vs WT-FC, n.s., WT-MC vs KO-FC, n.s.,KO-MC vs WT-FC p<0.001, KO-MC vs KO-FC p<0.001, WT-FC vs KO-FC,n.s. (Tukey's post test) |
|  | mature BDNF expression -male and female | Figure 3c | WT male cortex (WT-MC), KO male cortex (KO-MC), WT female cortex (WT-FC), KO female cortex (KO-FC) | 12, 16, 5, 7 | Two-way ANOVA: Sex effect P<0.0001, Gene effect P=0.0004 WT-MC vs. KO-MC, P <0.0001 , WT-FC vs. KO-FC, n.s. (Bonferroni post test) |
|  |  |  |  |  | One-way ANOVA, WT-MC vs KO-MC P<0.001, WT-MC vs WT-FC, n.s., WT-MC vs KO-FC, n.s.,KO-MC vs WT-FC P<0.001, KO-MC vs KO-FC P<0.001, WT-FC vs KO-FC, n.s. (Tukey's post test) |
|  | TrkB expression -male and female | Figure 3c | WT male cortex (WT-MC), KO male cortex (KO-MC), WT female cortex (WT-FC), KO female cortex (KO-FC) | 6, 4, 5, 6 | Two-way ANOVA: Sex effect P=0.0003, Gene effect P=0.0051 WT-MC vs. KO-MC, P <0.0001 , WT-FC vs. KO-FC, n.s. (Bonferroni post test) |
|  |  |  |  |  | One-way ANOVA, WT-MC vs KO-MC P<0.001, WT-MC vs WT-FC, n.s., WT-MC vs KO-FC, n.s.,KO-MC vs WT-FC, P<0.001, KO-MC vs KO-FC, P<0.001, WT-FC vs KO-FC, n.s. (Tukey's post test) |
|  | Shank3 expression | Figure 4c | WT-Cortex (WT-C),  KO-Cortex (KO-C) | 7, 7 | Mann Whitney test, P =0.0006, Unpaired t test with Welch's correction, P=0.0102 |
|  | MT1/2  expression |  | WT-Cortex (WT-C), KO-Cortex (KO-C) | 5, 4 | Mann Whitney test, P =0.0159, Unpaired t test with Welch's correction, P=0.0225 |
|  | BDNF expression | Figure 6d | Control (CTL), ClioQ (C), ClioQ+Zn (C+Z) | 3, 3, 3 | One-way ANOVA, CTL vs C, P<0.01, CTL vs C+Z, P<0.001, C vs C+Z, P<0.01 (Tukey's post test) |
|  |  | Figure 6f | Control (CTL), ClioQ+Zn (C+Z), CioQ+Zn+MC (C+Z+M) | 5, 5, 5 | One-way ANOVA, CTL vs C+Z, P<0.001, CTL vs C+Z+M, n.s., C+Z vs C+Z+M, P<0.01 (Tukey's post test) |
|  |  | Figure 7b | WT -Saline (WT-S), KO-Saline (KO-S), KO-Minocycline (KO-M) | 4, 8, 6 | One-way ANOVA, WT-S vs KO-S, P<0.001, WT-S vs KO-M, n.s., KO-S vs KO-M, P<0.001 (Tukey's post test) |
| Zymography | MMP-9 zymogram | Figure 4d | WT male(WT-M),  KO male (KO-M),  WT female (WT-F),  KO female (KO-F) | 7, 6, 4, 7 | Two-way ANOVA: Sex effect P=0.0043, Gene effect P=0.0054 WT-M vs. KO-M, P <0.001 , WT-F vs. KO-F, n.s. (Bonferroni post test) |
|  |  |  |  |  | One-way ANOVA, WT-M vs. KO-M, P<0.001, WT-M vs. WT-F, n.s.,WT-M vs. KO-F,n.s., KO-M vs. WT-F, P<0.01, KO-M vs. KO-F, P<0.001, WT-F vs. KO-F, n.s. (Tukey's post test) |
|  |  | Figure 6c | Control (CTL), ClioQ (C), ClioQ+Zn (C+Z) | 7, 6, 5 | One-way ANOVA, CTL vs C+Z, P<0.05, CTL vs C, n.s., C vs C+Z, n.s. (Tukey's post test) |
|  |  | Figure 6f | Control (CTL), ClioQ+Zn (C+Z), CioQ+Zn+MC (C+Z+M) | 5, 4, 4 | One-way ANOVA, CTL vs C+Z, P<0.01, CTL vs C+Z+M, P<0.05, C+Z vs C+Z+M, P<0.01 (Tukey's post test) |
|  |  | Figure 7a | WT -Saline (WT-S),  KO-Saline (KO-S), KO-Minocycline (KO-M) | 6, 7, 3 | One-way ANOVA, WT-S vs KO-S, P<0.05, WT-S vs KO-M, n.s., KO-S vs KO-M, P<0.05 (Tukey's post test) |
| Immunocyto-chemistry | Neurite number | Figure 5b | WT Neuron (WT-N), KO neuron (KO-N) | 7, 8 | Mann Whitney test, P=0.0003, Unpaired t test with Welch's correction, P<0.0001 |
|  | BDNF expression | Figure 5b | WT Neuron (WT-N), KO neuron (KO-N) | 8, 10 | Mann Whitney test, P<0.0001, Unpaired t test with Welch's correction, P<0.0001 |

**
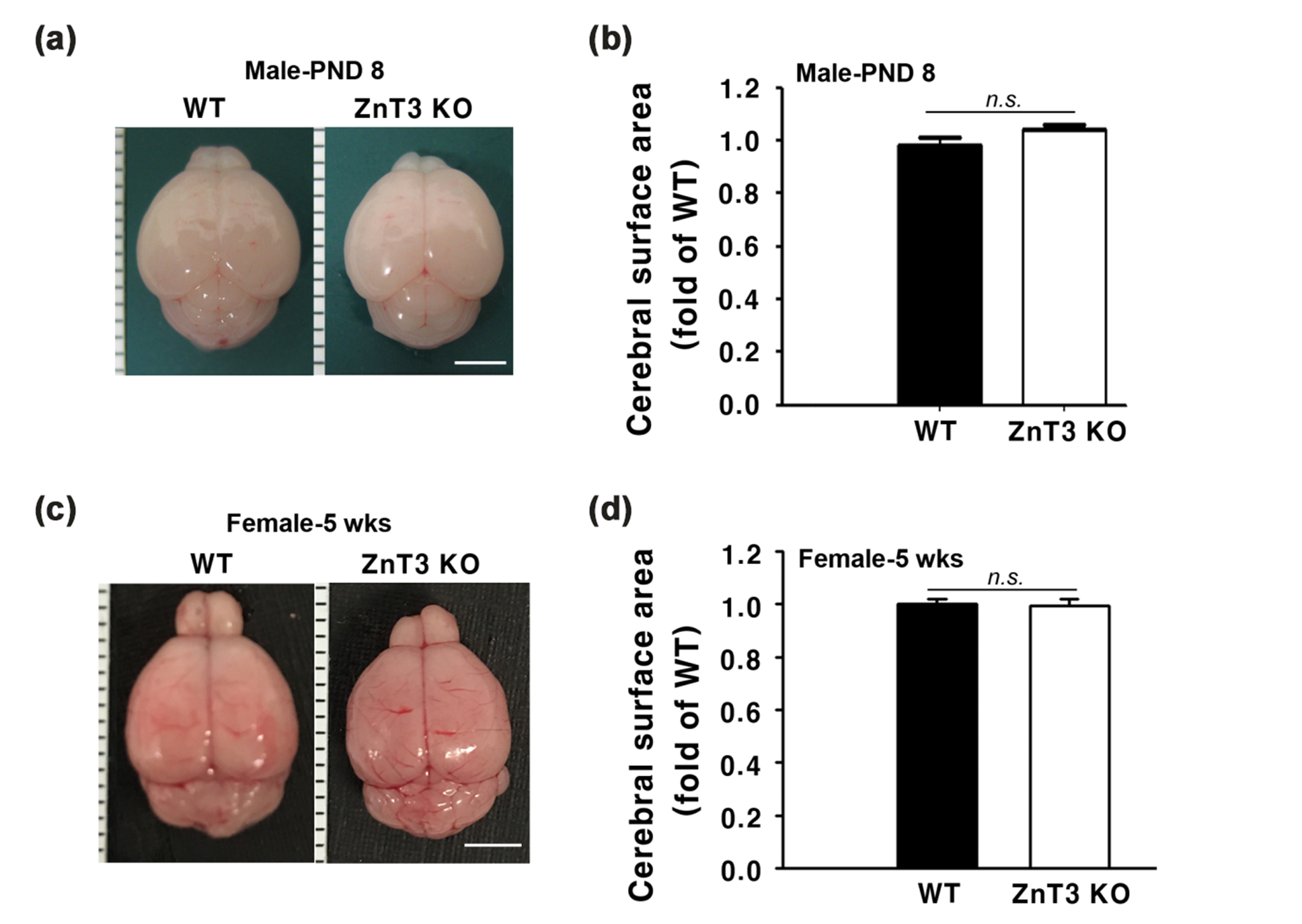
**

**Supplementary Figure S1. Comparison brains of WT and ZnT3 null mice.**

**(a)** Photographs of representative brains from male WT and ZnT3 mice at PND8. Scale bars represent 3 mm.

**(d)** Bars indicate the fold increase in cerebral surface as the area measured on an image analyzer (mean ± SEM, WT, n=5; KO, n=5, n.s.; *p*>0.05; Unpaired t-test with Welch’s correction).

**(c)** Photographs of representative brains from female WT and ZnT3 mice at 5 weeks of age. Scale bars represent 3 mm.

**(d)** Bars indicate the fold increase in cerebral surface as the area measured on an image analyzer (mean ± SEM, WT, n=7; KO, n=14, n.s.; *p*>0.05; Unpaired t-test with Welch’s correction).


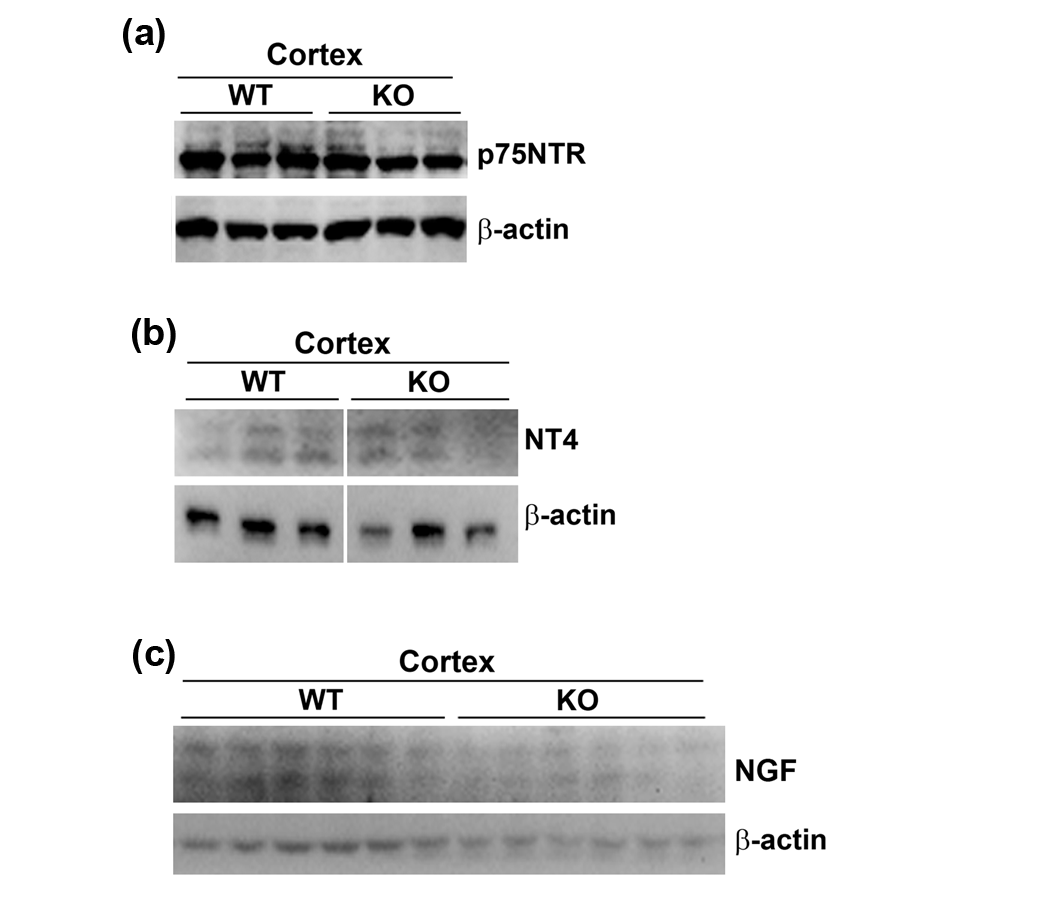


**Supplementary Figure S2. No changed to p75NTR, NT4 and NGF levels in male ZnT3 null brains.**

(**a**) Western blots of samples prepared from hippocampus and neocortex of male WT and ZnT3 null mouse brains with antibodies to p75NTR and β-actin. Levels of p75NTR were not altered in male ZnT3 null mouse brains (WT, n=3; KO, n=3).

(**b**) Western blots analysis for NT4 expression of neocortex in male WT and ZnT3 null mice. Levels of NT4 were not altered in male ZnT3 null mouse brains (WT, n=3; KO, n=3).

(**c**) Expression of NGF of neocortex in male and ZnT3 null brains. No changed expression of NGF in male ZnT3 null mouse (WT, n=6; KO, n=6).


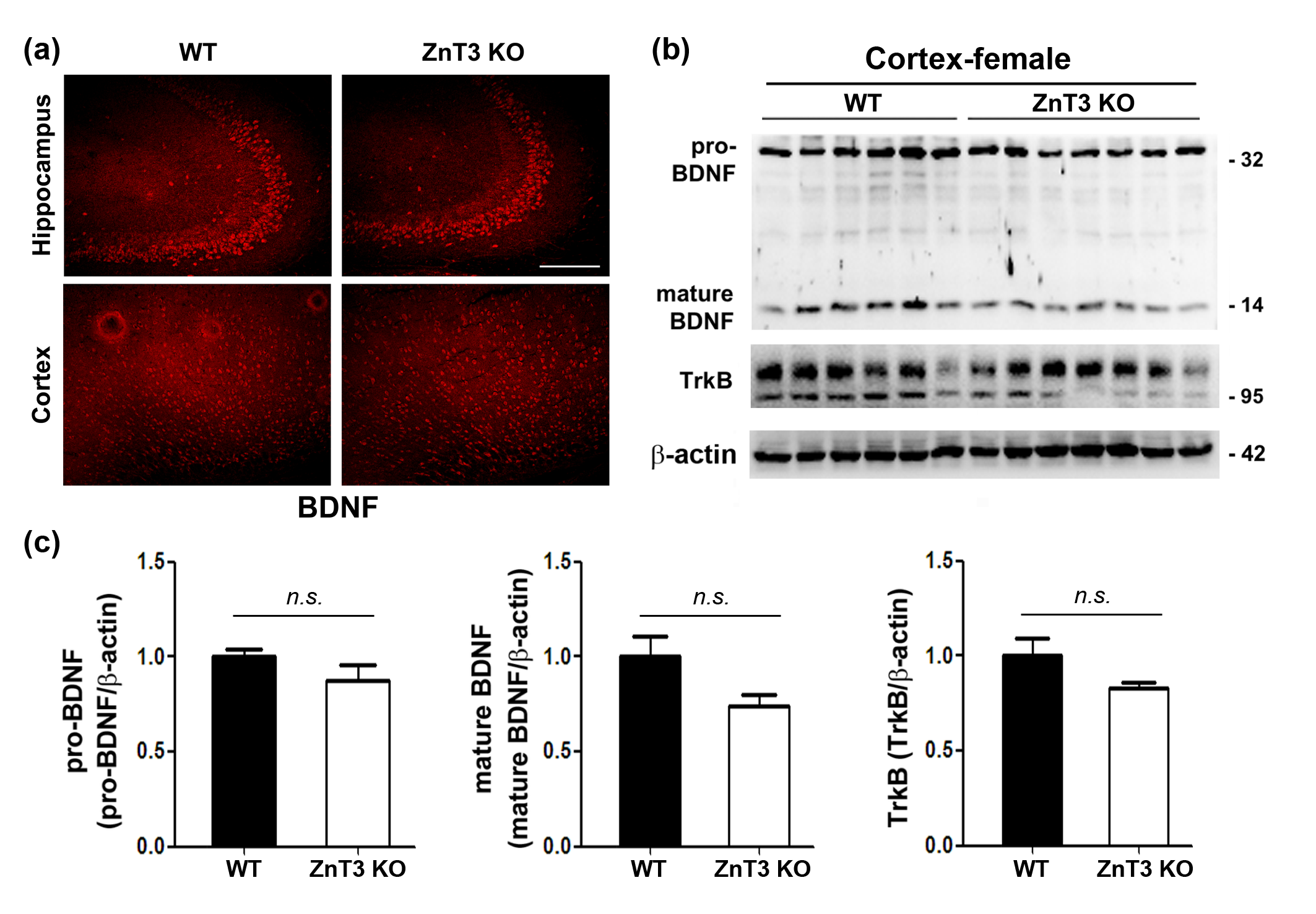


**Supplementary Figure S3. No changed BDNF and TrkB levels in female ZnT3 null mouse brains.**

(**a**) Fluorescence confocal photomicrographs of brains from female WT and ZnT3 null mice that were immunohistochemically stained for BDNF. BDNF immunoreactivity was not changed in ZnT3 null brains. Scale bar represents 200 μm.

(**b**) Western blots of samples prepared from the neocortex of female WT and ZnT3 null mouse brains analyzed with antibodies to BDNF, TrkB, and β-actin.

(**c**) Bars denote fold increases in the density of bands for pro-BDNF, mature BDNF, and TrkB in the cortex. No changed in pro-, mature BDNF and TrkB levels in female ZnT3 null brains (mean ± SEM, mature BDNF: WT, n=6; KO, n=7, n.s.: *p*>0.05; Two-way ANOVA with Bonferroni’s *post hoc* test with male samples).

**
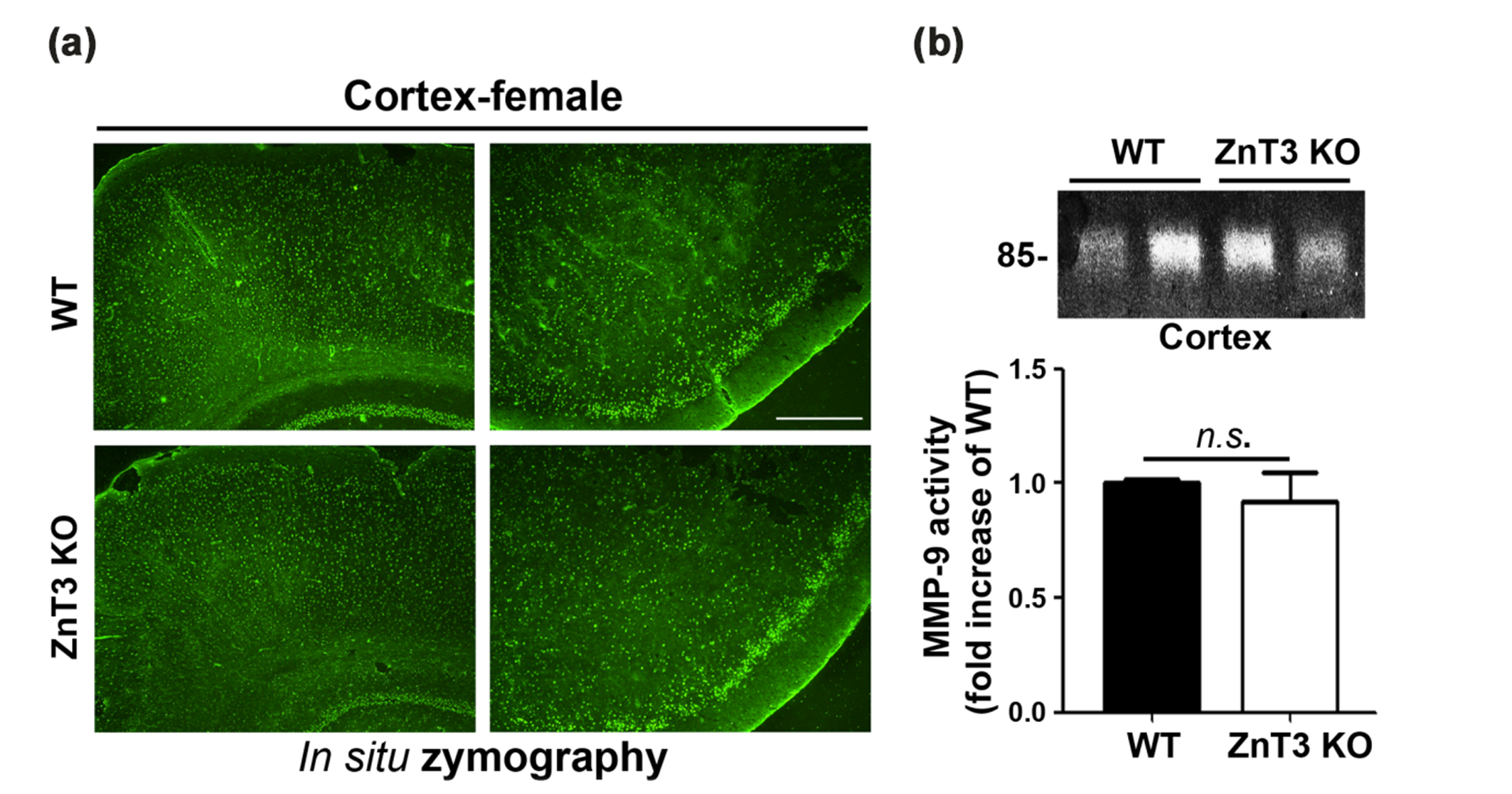
**

**Supplementary Figure S4. No changed MMP activity in female ZnT3 null mouse brains.**

(**a**) Fluorescence photomicrographs for MMP activity in cortex of female WT and ZnT3 null brains. No changed MMP (+) cells between WT and ZnT3 null cortex. Scale bar represents 200 μm.

(**b**) MMP-9 zymogram of proteins from female WT and ZnT3 brains. Samples from ZnT3 null brains showed no increased MMP activity compared with it of WT. Bars denote no differences in MMP-9 activity between female WT and ZnT3 null brains (mean ± SEM, WT, n=4, KO, n=7, n.s. *p*>0.05; Unpaired t-test with Welch’s correction).


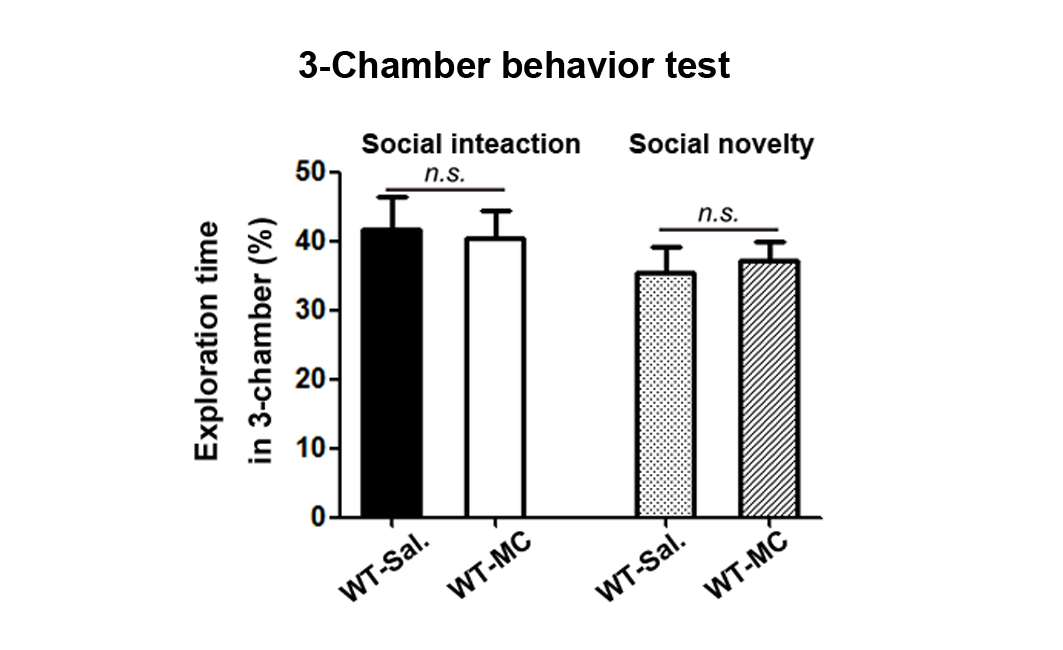


**Supplementary Figure S5. Minocycline administration did not change behaviors in wild type mice.**

Bars represent time spent with a stranger 1 (social interaction) or with a stranger 2 (social novelty) in the 3-chamber test. There were no differences in behaviors between saline-treated WT and minocycline-treated WT mice. (mean ± SEM, WT male-Sal, n=11; WT male-MC, n=9 *n.s*.: *p*>0.05; Two-way ANOVA with Bonferroni’s *post hoc* test).


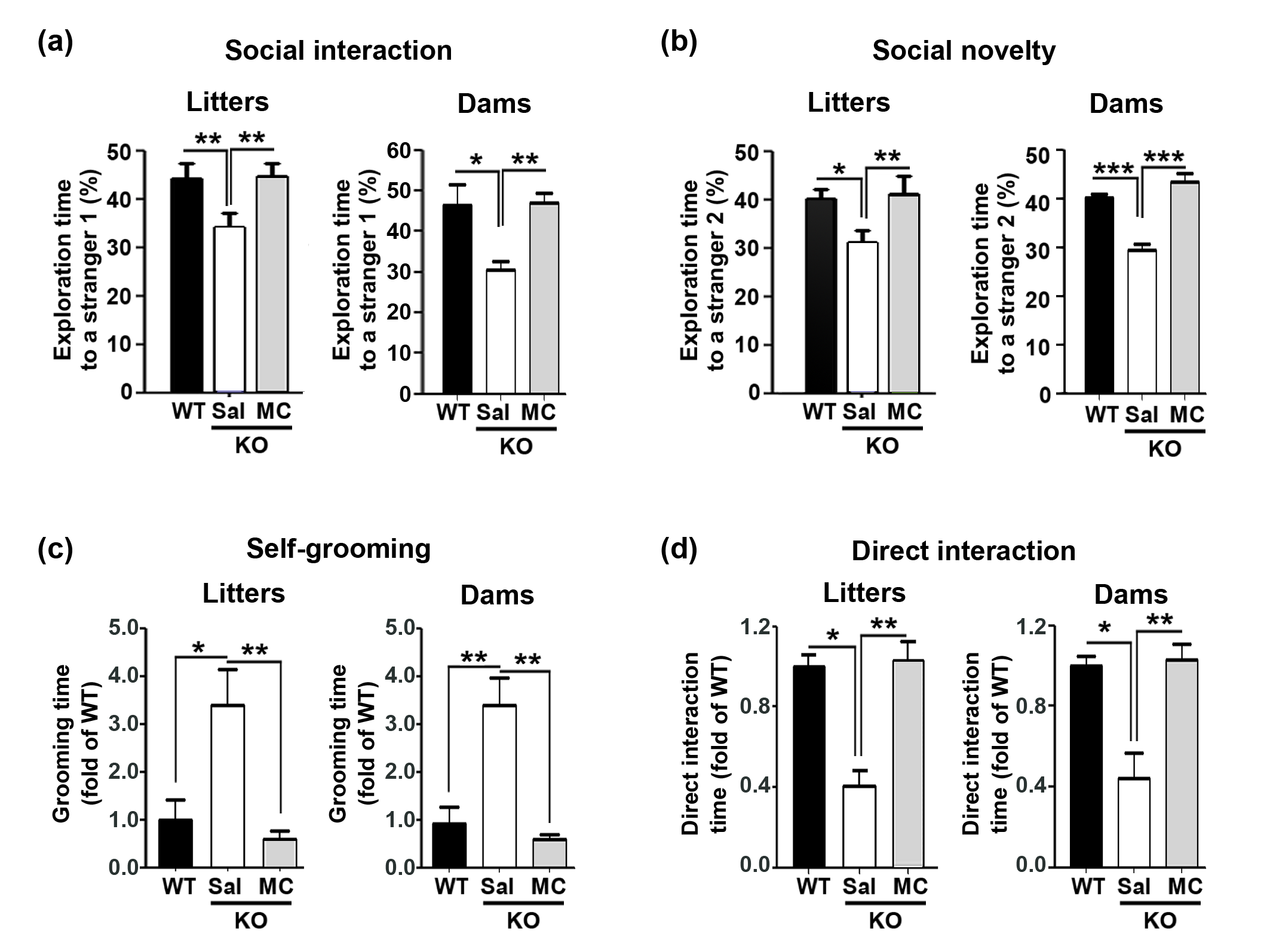


**Supplementary Figure S6. Minocycline administration did not have litter-specific effect in ZnT3 null mice.**

(**a**) Bars represent time spent with a stranger 1 with all litters (left) and with litters based on dams (right) in the 3-chamber test. The interaction with stranger 1 was greater in 50 mg/kg MC-treated ZnT3 null mice than in saline-treated ZnT3 null mice without litter-specific effect, because the pattern and statistics were not big different between two bar graphs (mean ± SEM, WT litters, n=19; KO litters-Saline, n=27; KO litters-MC, n=14, WT dams, n=5; KO dams-Saline, n=6; KO dams-MC, n=6, **p*<0.05, ***p*<0.01; one-way ANOVA with Tukey’s *post hoc* test).

(**b**) Bars represent time spent with a stranger 2 with all litters (left) and with litters based on dams (right) in the 3-chamber test. The interaction with stranger 2 was greater in the MC-treated group than in the saline treated ZnT3 null mice group with no litter-specific effect (mean ± SEM, WT litters, n=19; KO litters-Saline, n=27; KO litters-MC, n=14, WT dams, n=5; KO dams-Saline, n=6; KO dams-MC, n=6, **p*<0.05, ***p*<0.01, ****p*<0.0001; one-way ANOVA with Tukey’s *post hoc* test).

(**c**) Bars represent time spent on self-grooming in the 3-chamber test. Self-grooming of ZnT3 null mice was significantly increased compared with WT mice and it was reduced by MC administration without litter-specific effects (mean ± SEM, WT litters, n=7; KO litters-Saline, n=12; KO litters-MC, n=8, WT dams, n=3; KO dams-Saline, n=4; KO dams-MC, n=4, **p*<0.05, ***p*<0.01; one-way ANOVA with Tukey’s *post hoc* test).

(**d**) Bars represent time spent in direct interaction with stranger 1 mouse in the 3-chamber test. The direct interaction time was greater in MC-treated ZnT3 null mice than in saline-treated ZnT3 null mice without litter-specific effects (mean ± SEM, WT litters, n=6; KO litters-Saline, n=10; KO litters-MC, n=8, WT dams, n=3; KO dams-Saline, n=4; KO dams-MC, n=4, **p*<0.05, ***p*<0.01; one-way ANOVA with Tukey’s *post hoc* test).
